# Flexible Investment Casting Wax Patterns for 3D-Printing: Their Rheological and Mechanical Characterizations

**DOI:** 10.3390/polym14214744

**Published:** 2022-11-05

**Authors:** László Szabó, György Deák, Dávid Nyul, Sándor Kéki

**Affiliations:** 1 Bogdány Petrol Ltd., Gyártelep, H-4511 Nyírbogdány, Hungary; 2 Doctoral School of Chemistry, University of Debrecen, Egyetem tér 1, H-4032 Debrecen, Hungary; 3 Department of Applied Chemistry, Faculty of Sciences and Technology, University of Debrecen, Egyetem tér 1, H-4032 Debrecen, Hungary; 4 Department of Physical Chemistry, Faculty of Sciences and Technology, University of Debrecen, Egyetem tér 1, H-4032 Debrecen, Hungary

**Keywords:** investment casting wax, 3D printing, Piccotex 75, Escorene, rheology, mechanical properties

## Abstract

The mechanical and rheological characterizations of flexible investment casting patterns capable of 3D printing are reported. The wax pattern was composed of microcrystalline hydrocarbon wax (DMW7478), Piccotex 75 (a copolymer of α–methyl–styrene and vinyl toluene with a 75/25 molar ratio, respectively) and Escorene (a copolymer of ethylene and vinyl acetate with a 72/28 mass ratio, respectively). It was found that in order to obtain a wax pattern with appreciable mechanical properties, the content of the microcrystalline hydrocarbon wax in these blends should not exceed 30% (m/m). Thus, a series of patterns with 28% (m/m) wax and varying Piccotex and Escorene contents spanning from 0 to 72% (m/m) was prepared. The dependence of the dynamic viscosities of the wax patterns on the composition was described using a stretched exponential model, whereas their variations with the temperature were interpreted in terms of the Arrhenius–Guzman equation. Furthermore, the slopes of the lines fitted to the viscosity versus temperature curves at the pour point decreased linearly with the Piccotex content. Non-Newtonian changes in the shear stress with the shear rate and shear stress crystallization were observed at temperatures near the pour points. The mechanical properties were evaluated using the uniaxial tensile mode and by three-point bending experiments. It was found that the stress (σ) versus the relative elongation (ε) curves can effectively be rendered by means of the standard linear solid (SLS) viscoelastic model. In addition, it was also established that the Young’s modulus varied according to a sigmoid-type curve with the piccotex content, while the yield stress decreased linearly with the concentration of Piccotex. In addition, based on the spooling suitability and printability, the patterns were rated and it was found that the most appropriate wax pattern compositions for 3D printing were those which contained 30% (m/m) and 35% (m/m) Piccotex.

## 1. Introduction

Wax is one of the longest-used thermoplastic materials known in the history of humankind [1]. Because of its fluid/solid, malleable and workable properties, it has been tightly linked to various fields in our everyday lives [2]. However, currently, the term wax is used to describe all natural and synthetic materials whose properties are similar to those of beeswax [3]. Waxes are used mainly in foundries due to their variable and versatile properties that enable them to produce finished products that meet various requirements. For example, the metallic objects produced by the investment casting process are indispensable to automotive, aerospace, chemical and mechanical engineering industries, and their production either cannot or can insufficiently be replaced by other processes [4]. The main steps of investment casting, also referred to as the lost wax process, include coating the wax pattern with a series of layers of refractory slurry consisting of ceramic and binder materials to form a shell, followed by dewaxing and calcination. Then, the molten metal is poured into the dewaxed shell to take its shape, and after the solidification of the metal, the object is removed from the shell [5]. The most typical waxes used in the investment casting process are petroleum-based waxes (paraffin or microcrystalline hydrocarbons), natural and synthetic waxes, and their blends [6]. Waxes without fillers and/or any other components or ingredients have been widely used for a long time for the casting process, especially when the goal is to produce simple and small objects, e.g., for jewelry [7]. Moreover, the demand for the fabrication of more complex and bigger objects has led to the development of wax patterns with improved physical properties by using various fillers and/or blending the wax with polymeric materials [8]. Thus, the physical properties, such as the melting/softening point, hardness, viscosity, rheology, thermal expansion and shrinkage of wax-like materials, are all greatly influenced by their compositions [9]. Huge developments in plastic processing, such as the introduction and application of 3D printing in this area, have made it possible to produce more complex objects, which are not or may not be feasible via other processing technologies [10]. As for waxes, to the best of our knowledge, only a few reports, which for the most part are patents on 3D-printable waxes, have appeared. In these studies, petroleum waxes, beeswax, polyethylene wax, polymer resin and stearic acid were employed [11,12,13,14,15]. In another patent, three-dimensional printing wax materials, containing petroleum wax, animal wax, vegetable wax, mineral wax, synthetic wax, a viscosity regulator, a toughening agent, a hardness regulator, and sodium starch octenyl succinate, which is useful for jewelry, are described [16]. However, when utilizing the advantages of 3D-printing techniques [17] in investment casting wax technology to produce more complex metallic parts, we run into several difficulties: (i) waxes are generally fragile or at least not flexible enough to use them as filaments for 3D printing. (ii) The elastic modulus (Young’s modulus), yield stress and tensile strength are too low to introduce them into a 3D-printing machine without breaking. (iii) The temperature of the print head should be held at a relatively low temperature and carefully compensate for the fast cooling in order to avoid layering the wax pattern during 3D printing. (iv) The rheological properties of the unblended and unfilled wax are not suitable for 3D printing. (v) The finished wax pattern can reveal shrinkage after casting, printing and/or deformation during storage. Therefore, in order to obtain investment casting waxes suitable for 3D-printing technology, the mechanical and rheological properties should be modified to eliminate the deleterious effects outlined in (i)–(v). One of the simplest ways to have wax with appropriate properties for printing is to make blends that contain, in addition to hydrocarbon wax, (co)polymers that give flexibility and others that can provide shape fixity to the wax patterns. 

In our present study, we report our detailed mechanical and rheological investigations of the blends of a microcrystalline with Piccotex 75 (a copolymer of α–methyl–styrene and vinyl toluene with a 75/25 molar ratio, respectively) and Escorene (a copolymer of ethylene and vinyl acetate with a 72/28 mass ratio, respectively). Furthermore, it is shown that appropriate wax pattern compositions for 3D printing can be made using microcrystalline hydrocarbon wax, Piccotex and Escorene. 

## 2. Experimental

### 2.1. Chemicals

Microcrystalline hydrocarbon wax DMW7478 with pour points between 74 °C and 78 °C was obtained from MOL plc. (Szászhalombatta, Hungary). The gas chromatographic (GC) trace of DMW7478 is shown in Figure S1 (Supporting Information). Piccotex 75 (a copolymer of α–methyl–styrene and vinyl toluene with a 75/25 molar ratio, respectively) was received from Eastman Chemical Co. (Kingsport, TN, USA). The matrix-assisted laser desorption/ionization mass spectrum (MALDI–MS) of Piccotex 75 is exhibited in Figure S2 (Supporting Information). Escorene UL 40028 CC (a copolymer of ethylene and vinyl acetate with a 72/28 mass ratio, respectively) was purchased from ExxonMobil Chemical Co. (Spring, TX, USA).

### 2.2. Sample Preparation

A Teflon-coated metal pot was used to prepare the wax blends in 500 g quantities for rheological and mechanical testing. A typical procedure for blending is presented below. 

To 140 g of the melted hydrocarbon wax, 185 g granules of ethylene–vinyl acetate copolymer (Escorene UL 40028 CC) were added and stirred using a KPG stirrer until dissolution. The temperature was kept at 150 °C–180 °C. It was found that granules in the molten state were present for a long time with an almost water-like, clear appearance and dissolved completely after 15–20 min. After complete dissolution, 175 g of Piccotex 75 was added and the blends were continuously stirred further. The homogeneous melted blends were poured into silicone molds with sizes of 10 cm × 5 cm × 2.5 cm afterwards. The compositions of wax samples prepared are summarized in Table 1.

The three-component samples listed in Table 1 were homogeneous when melted and remained homogeneous after cooling and solidifying in the silicon mold.

### 2.3. Instrumental Methods

*Filament Extrusion*

A single-screw extruder (Göttfert G20, Buchen, Germany) with three-zone temperature connected to a Brabender conveyor belt was used to make filaments for 3D printing. The main parameters of the extruder were as follows: zone temperatures: 50 °C, 55 °C and 60 °C; screw rotation speed: 16 rpm; screw compression ratio: 1/4; pressure: 30 bar; filament collecting speed: 5 m/min; and collected filament diameter: 1.75 mm.

*3D Printing*

The 3D printer used in this study was an Anet MK8 (Shenzhen Anet Technology Co., Ltd., Xinqiao, China). The following printing conditions were used: the printing speed was maintained at 60 mm/s using a print head temperature of 120 °C and print bed temperature of 25 °C. The layer height was set to 0.2 mm. A nozzle with a diameter of 0.4 mm and a wall thickness of 1.6 mm was used. 

*Rheological Measurements*

The rheology studies were performed with a rotational rheometer from Malvern, UK (Kinexus Pro) equipped with a 4 cm stainless-steel plate. All the rheological measurements were carried out in the steady-state mode using the plate geometry. The shear stresses were recorded with the range of shear rate from 1 s^−1^ to 150 s^−1^ using a ramp time of 2.5 min. For viscosity versus temperature measurements in the temperature range of 60 °C–120 °C, a shear rate of 10 s^−1^ and temperature ramp of 2 °C/min were employed. We used the shear rate of 10 s^−1^ based on a recommendation from T. Mezger, which is a local laboratory standard for the investigation of such kinds of waxes [18].

*Tensile Measurements*

An Instron 4302 mechanical testing machine (Instron, Norwood, MA, USA), equipped with a 1 kN load cell, was used for tensile testing of the samples. The samples were tested according to EN ISO 527–1 standard. Five dumbbell specimens were cut (clamped length 40 mm) from the samples and the thickness and width of the samples were 1 and 6 mm, respectively. The crosshead speed was 5 mm/min. The limit of displacement was set to 80 mm, or break, whichever came first. A 10 pts/s sampling rate was used in all cases. For the calculations of the Young’s modulus, yield stress, yield strain, stress at break and strain at break values, an Instron series 9 Automated Materials Tester with version 8.30.00 software (Instron, Norwood, MA, USA) was used.

*Flexural Measurements*

The same instrument (Instron 4302) was used as in the tensile tests modified for three-point bending measurements. The tests were performed according to EN ISO 178 standard. Length of span was 60 mm, and crosshead speed was 2 mm/min. The thickness, width and length of the samples were 4, 10 and 100 mm, respectively. Five samples were tested and the flexural stress (Equation (1)), flexural strain (Equation (2)) and flexural modulus (Equation (3)) were calculated.

Flexural stress:

(1) $$\sigma_{f}=\frac{3\mathrm{FL}}{{2\mathrm{bh}}^{2}}$$

where σ_f_ is the flexural stress; F is the applied force in Newton; and L, b and h are the span in millimeters, the width in millimeters of the specimen and the thickness in millimeters, respectively.

Flexural strain:

(2) $$\varepsilon_{f}=6\mathrm{sh}/L^{2}$$

where ε_f_, s, h and L are the flexural strain parameter (dimensionless), the deflection in millimeters, the thickness of the specimen in millimeters and the span in millimeters, respectively.

Flexural modulus:

(3) $$E_{f}=\frac{\sigma_{f2}-\sigma_{f1}}{\varepsilon_{f2}-\varepsilon_{f1}}$$

where E_f_, σ_f2_ and σ_f1_ are the flexural modulus in MPa, the flexural stress at ε_f2_ in MPa, and the flexural stress at ε_f1_ in MPa, respectively, and ε_f2_ = 0.0025 and ε_f1_ = 0.0005.

## 3. Results and Discussion

### 3.1. Rheological Properties

**Figure 1:**
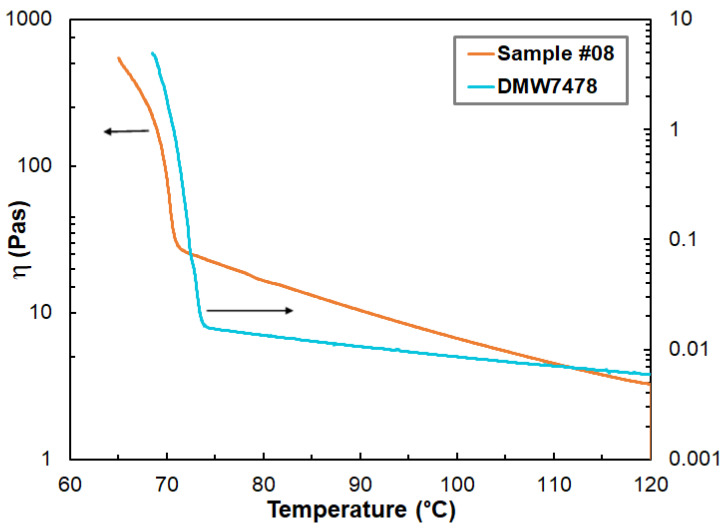
Variation in the dynamic viscosities with temperature and compositions for pure microcrystalline wax DMW7478 and sample #08. The shear rate was 10 s^−1^. The arrows show the corresponding axis.

In terms of practical considerations, microcrystalline petroleum-based waxes with pour points between 74 °C and 78 °C were used. The reason behind this was mainly that hydrocarbon waxes with around these pour points are frequently used in the investment casting process [19]. Furthermore, waxes with lower pour points would otherwise be too sensitive to higher temperatures, resulting in shape deformation. As it was pointed out in the Introduction, for waxes capable of 3D printing, some important mechanical and rheological requirements should be fulfilled. Therefore, in order to improve the mechanical and rheological properties for 3D printing, blends of hydrocarbon wax DMW7478, Escorene and Piccotex 75 were prepared in different compositions, and the rheological properties of the resulting blends were systematically investigated. According to the preliminary experiments, the hydrocarbon wax content in the blends should not exceed 30% (m/m), since at higher concentrations, the viscosity of the mixture was very low and the duration of the plastic phase after the pour point was too short for extrusion. In addition, the mechanical properties of the resulting blends with a higher wax content were insufficient for 3D printing. Thus, hydrocarbon wax contents of 28% (m/m) were applied, the Piccotex 75 content varied from 0 to 72% (m/m) and the Escorene content simultaneously spanned from 72% (m/m) to 0% (m/m). As representative examples, Figure 1 shows the change in the dynamic viscosity at a constant shear rate as a function of the temperature for the hydrocarbon wax and sample #08. 

As seen in Figure 1, the viscosity of the hydrocarbon wax in the range of 75 °C–120 °C is much lower than those of the blends, which can be attributed to the viscosity-increasing properties of Escorene and Piccotex. Furthermore, as it turns out from the data in Table 2, the pour point of DMW7478 is at 74.1 °C and the presence of the polymers Escorene and Piccotex has only a minor effect on the resulting pour points. Furthermore, the pour points of the blends are lower than that of the pure DMW7478 wax, and despite the wide composition range, the values of the pour points of the blends remain within a narrow temperature range (69.5 °C–72.8 °C). Interestingly, copolymers based on poly (ethylene–co–vinyl acetate)-containing hydroxyl groups and long-pendant hydrocarbon chains (from C_6_ to C_18_) greatly reduced the pour point of the crude oil, thereby preventing the precipitation of the wax [20]. In our case, however, no such effect was observed, probably due to the lack of long carbon chains and hydroxyl groups. Thus, the absence of considerable pour point depression is advantageous in our case, since the blends retain their pour points close to that of the pure hydrocarbon wax, independent from the compositions. 

**Figure 2:**
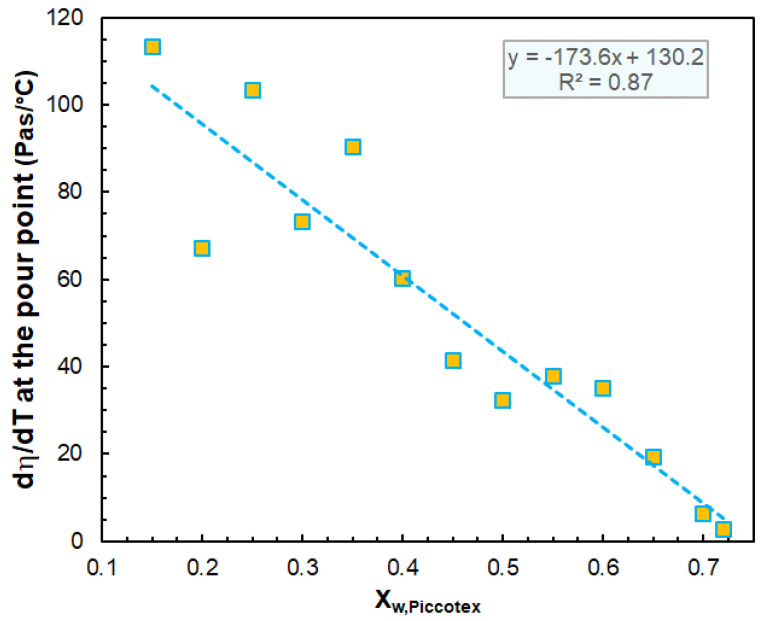
Variation in the dη/dT values at the pour point with the Piccotex content. The shear rate was 10 s^−1^.

**Figure 3:**
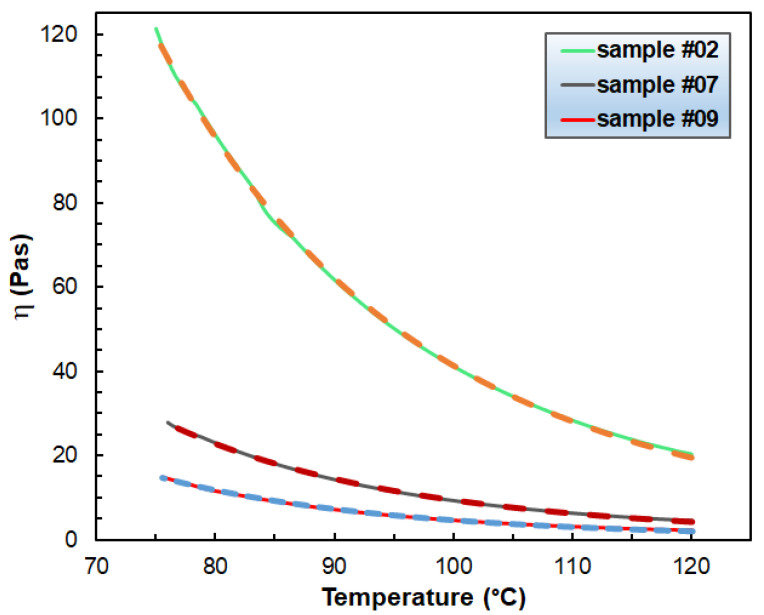
The dependence of the viscosity on the temperature in the range of 75 °C –120 °C for samples #02, #07 and #09. The solid and the dashed lines stand for the measured and fitted (via Equation (4)) curves. The shear rate was 10 s^−1^. The fitted parameters are A = 1.65 × 10^−5^, 1.40 × 10^−6^ and 4.10 × 10^−7^ Pas, and B = 5498, 5865 and 6062 K for samples #02, #07 and #09, respectively.

It is worth investigating the value of dη/T of the viscosity versus temperature curves at the pour point. Figure 2 exhibits the variation in the dη/dT values at the pour point with the Piccotex content. As Figure 2 demonstrates, the dη/dT values vary closely and linearly with the Piccotex content. This also indicates that in this three-component system with a constant hydrocarbon wax content, the Escorene predominantly determines the dη/dT values. On the other hand, as emphasized before, these values may have a significant role in designing and tailoring the extrusion process. The dependencies of the viscosity on the temperature in the range of 75 °C–120 °C obey the Arrhenius–Guzman equation (Equation (4)) [21], as demonstrated in Figure 3.

(4) $$\eta (T)={\mathrm{Ae}}^{B/T}$$

where A is the pre-exponential factor and B = E_η_/R, E_η_ and R are the activation energy and the universal gas constant, respectively.

**Figure 4:**
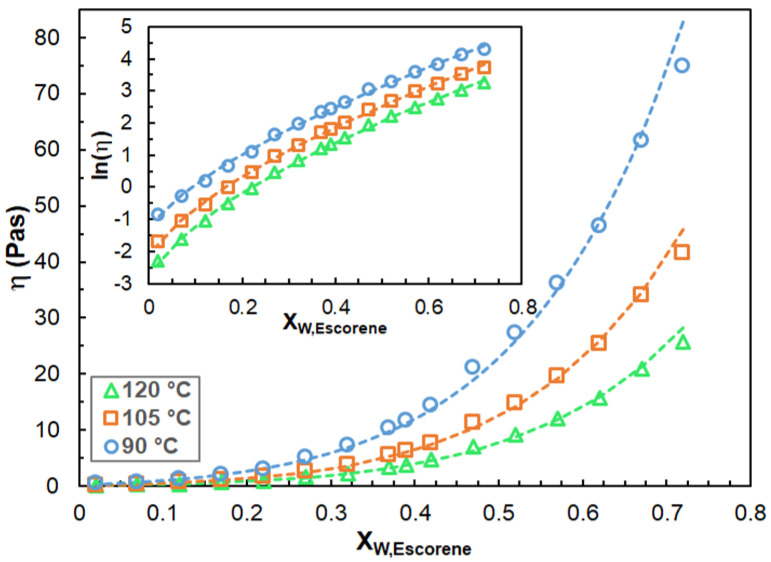
The viscosity versus weight fraction of Escorene at temperatures of 90 °C, 105 °C and 120 °C. The inset shows the ln(η) versus X_w,Escorene_ curves. The symbols and the dashed lines stand for the experimental and the fitted data (via Equation (5)), respectively. The fitted parameters are: α = 0.045, 0.089, 0.230; β = 28.5, 24.3, 19.3; and *γ* = 0.616, 0.640, 0.673. The shear rate was 10 s^−1^.

As it turns out from Figure 3, the curves fitted by Equation (4) match the experimental ones perfectly and this finding is also true for the rest of the samples. The fitted parameters are summarized in Table S1 in the Supporting Information. It is also important to evaluate how the viscosity varies with the blend composition. In Figure 4, the viscosity as a function of the weight fraction of Escorene at three different temperatures is plotted. As seen in Figure 4, the dynamic viscosities increase considerably with the Escorene content. 

It was found that the experimental η–X_w,Escorene_ curves can adequately be described at all temperatures by a stretched exponential model, as shown in Equation (5).

(5) $$\eta ={\alpha e}^{{(\beta X_{W,\mathrm{Escorene}})}^{\gamma }}$$

where α, β and γ are the fitting parameters.

As it can be discerned from Figure 4, the proposed stretched exponential model renders the experimental η data adequately and this model is also capable of describing the η–X_w,Escorene_ relationships with a wide range of X_w,Escorene_ values. Interestingly, in other chemical systems, single exponential relationships were found between the viscosity and composition, such as in the case of the dimer–trimers of hexamethylene–diisocyanate [22] and the terner system of propylene glycol, glycerol and water [23]. 

**Figure 5:**
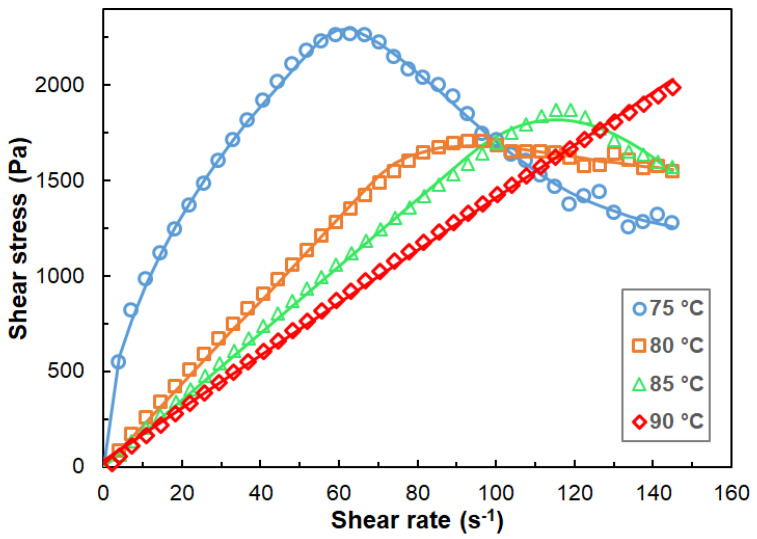
Shear stress versus shear rate curves for sample #07 at 75 °C, 80 °C, 85 °C and 90 °C. The symbols and solid lines represent the experimental and the fitted curves via Equations (7)–(9). The fitted parameters are as follows: 75 °C: a = 3.36 × 10^−4^, b = 3.95 × 10^−4^, m = 0.54, c_1_ = 276.1, n = 0.52, c_2_ = 126.2 and p = 0.40; 80 °C: a = 2.43 × 10^−3^, b = 2.35 × 10^−4^, m = 0.61, c_1_ = 21.8, n = 1.0, c_2_ = 9.7 and p = 0.18; 85 °C: a = 3.10 × 10^−3^, b = 4.32 × 10^−5^, m = 1.15, c_1_ = 17.5, n = 1.0, c_2_ = 2.0 and p = 1.0; 90 °C: c_1_ = 17.5, n = 1.0, c_2_ = 2.0; and all the other parameters are 0.

The dependence of the shear stress on the shear rate was also studied. As a representative example, the shear stress versus shear rate curves for sample #07 recorded at different temperatures are plotted in Figure 5.

As seen in Figure 5, the shear stress (τ)–shear rate (dγ/dt) curves change significantly with the temperatures. At a temperature close to the pour point, a relatively sharp, non-linear increase in the shear stress with the shear rate can be observed (at 75 °C). After reaching a maximum, the shear stress starts to decrease with the shear rate. τ–dγ/dt curves with a similar shape were also reported, for example, for the waxy crude oils [24], asphalt binders [25] and clay slurry with different solid volumes [26]. Moreover, in our case, increasing the temperature further (to 80 °C and 85 °C), the shear stress changes linearly with the shear rate (i.e., behaves as a Newtonian fluid) until the maximum. At higher temperatures (at 90 °C), no decrease in the shear stress can be recognized and the shear stress depends linearly on the shear rate, indicating a Newtonian fluid property in the shear range of 1 s^−1^–160 s^−1^. A similar trend can be observed for the rest of the samples as well. Furthermore, it was found that with the increasing Piccotex content, i.e., with the decreasing Escorene content, the shear stress versus shear rate curves became linear, even at temperatures close to the pour point in the case of blends with low Escorene contents. On the contrary, at temperatures close to the pour point for blends with high Escorene contents, the shear stress–shear rate curves obey the Ostwald–de Waele equation (Equation (6)) with a power of n < 1 (non–Newtonian, shear thinning fluid with pseudoplasticity). The shear stress–shear rate curves for samples #01–#16 recorded at 90 °C and the viscosity versus shear rate curves for sample #07 in the temperature range of 75 °C–110 °C are presented in Figures S3–S14 in the Supporting Information, respectively.

(6) $$\tau =c_{1}{\left(\frac{d\gamma }{\mathrm{dt}}\right)}^{n}$$

where c_1_ is a constant, a measure of the consistency of the fluid.

Judging from the shape of the shear stress–shear rate curves, it can also be surmised that in these three-component systems, shear-stress-induced crystallization may take place at higher shear rates. Indeed, inspecting the samples, e.g., sample #07 at 75 °C at different shear rates, it can be observed that the homogeneous and transparent mixture became opaque after the maximum, i.e., at a shear rate higher than 60 s^−1^ (Figure S15). Furthermore, in line with this observation, in the case of the samples that showed no maximum as a function of the shear rates, the mixture remained clear over the entire shear rate range investigated. In addition, it turned out that when decreasing the Escorene content, the blends tended to behave as a Newtonian fluid. For example, in the case of sample #16, only a small deviation from the Newtonian property can be observed at 75 °C, i.e., near the pour point (Figure S8). Note that sample #01 contains Escorene, while sample #16 only contains Piccotex in addition to the DMW7478 wax. Thus, it can be concluded from this finding that the interaction leading to crystallization or co-crystallization takes place primarily between the DMW7478 wax and the Escorene, which is, as mentioned before, an ethylene–vinyl acetate (EVA) copolymer. Interestingly, the interaction of wax in crude oil with an EVA copolymer is believed to be the main reason for the depressant effect on the pour point of the EVA copolymers [27,28]. 

To simulate and describe the τ–dγ/dt curves formally, we propose the application of the Houska model [29,30] (which was invented primarily for the description of the thixotropic effect) to our three-component systems, as shown by Equations (7) and (8).

(7) $$\frac{d\chi }{\mathrm{dt}}=k_{d}\chi -k_{c}(1-\chi ){\left(\frac{d\gamma }{\mathrm{dt}}\right)}^{m}\;\mathrm{if}\;d\gamma /\mathrm{dt}\;>\;(d\gamma /\mathrm{dt}{)}_{c}$$

χ = 0    if dγ/dt < (dγ/dt)_c_ (8)

where χ is the fraction of the crystalline phase, k_d_ and k_c_ are the rate constants for the dissolution and formation of the crystalline phase, and m is the power of the shear rate. 

Equations (7) and (8) express that if the shear rate is higher than a critical value ((dγ/dt)_c_), the molecules start to organize themselves into crystals, whereas if (dγ/dt)_c_ is lower, then no crystallization takes place.

The total shear stress has been taken to be a combination of the stress imposed by the non-crystalline and crystalline phases, as revealed by the first and second terms of Equation (9), respectively.

(9) $$\tau =(1-\chi {)c}_{1}{\left(\frac{d\gamma }{\mathrm{dt}}\right)}^{n}+\chi c_{2}{\left(\frac{d\gamma }{\mathrm{dt}}\right)}^{p}$$

where c_1_ and c_2_ as well as n and p are the parameters to be fitted to the experimental data.

By means of Equations (7)–(9), the corresponding parameters can be estimated. The c_1_ and m parameters of Equation (9) can be determined using the range of the shear rate up to the maximum shear stress, since in this region, it is supposed that no considerable amount of crystals was formed, allowing us to estimate the values of c_1_ and m. As it can be recognized in Figure 5, very good fittings to the experimental data were obtained. It is to be noted, however, that in order to validate the model, additional independent experiments need to be performed, e.g., to establish the cross-correlation between the parameters of the proposed model (if any) (Equations (7)–(9)).

### 3.2. Mechanical Properties

The mechanical properties play a significant role in designing 3D-printable wax patterns; therefore, the Young’s modulus, yield stress, yield strain, stress at break, strain at break and flexural modulus were determined for samples #01–#16 and the corresponding results are compiled in Table 3.

**Figure 6:**
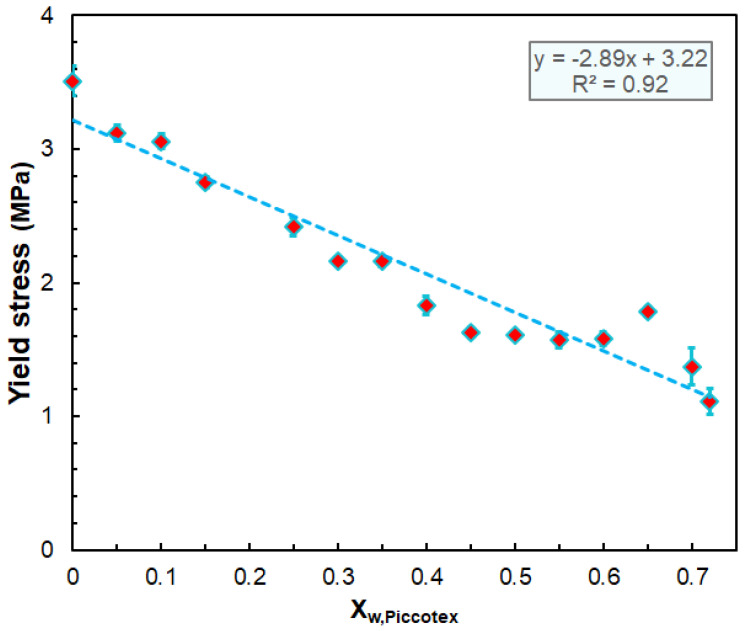
Variation in the yield stress with the weight fraction of Piccotex.

As expected, according to the data of Table 3, the mechanical properties are influenced by the composition of the blend. As a general tendency, it can be established that the yield stress decreases linearly with the Piccotex content, as also shown by Figure 6.

**Figure 7:**
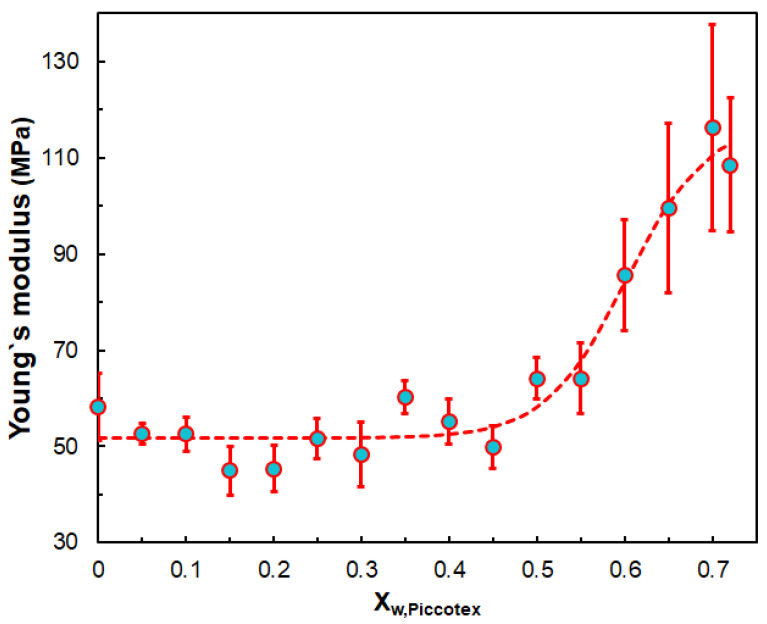
Variation in the Young’s modulus with the weight fraction of Piccotex. The dashed curve is the fitted curve via Equation (10). The fitted parameters are as follows: d_1_ = 51.8 MPa, d_2_ = 65.7 MPa, d_3_ = 60.1 and d_4_ = 4.6.

The stress at break also decreases with the Piccotex weight fraction but not in a linear manner. The findings that both the yield stress and the stress at break decrease with the Piccotex content (i.e., with the increase in the Escorene content) can be attributed to the more pronounced viscoelastic property of Escorene over Piccotex. Furthermore, a straightforward relationship cannot be established between the strain at break and stress at break; however, the yield strain increases with the yield stress until a value of ca. 2.7 MPa is reached, and after that, the strains level off at about 30%. The obtained Young’s modulus and flexural modulus values are also in good agreement. Interestingly, the Young’s modulus does not change significantly with the Piccotex content up to X_w,Piccotex_ = 0.45; however, at higher X_w,Piccotex_ values, a considerable increase in the values of the Young’s modulus can be attained, as demonstrated in Figure 7.

The dependence of the Young’s modulus on the Piccotex content can be characterized by a four-parameter sigmoid model (Equation (10)).

(10) $$E=d_{1}+\frac{d_{2}}{1+\exp\left[-\frac{{(X}_{W,\mathrm{Piccotex}-d_{3}})}{d_{4}}\right]}$$

where d_1_, d_2_, d_3_ and d_4_ are the parameters of the sigmoid function characterizing a hypothetical distribution.

A similar shape, but not a mathematically analyzed relationship, can also be established, e.g., for the impact strength versus methyl methacrylate–co–butadiene–styrene copolymer content in the case of poly (lactic acid)/methyl methacrylate–butadiene–styrene copolymer blends [31]. 

**Figure 8:**
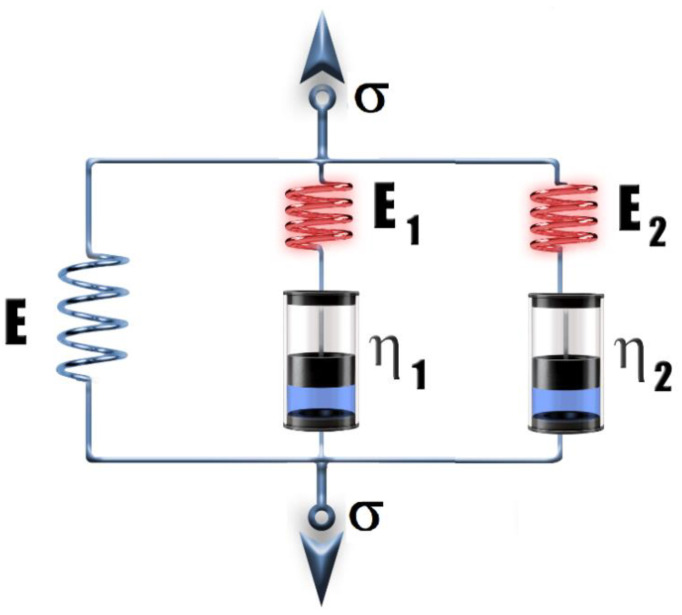
Viscoelastic standard linear solid (SLS) model extended with an additional Maxwell element (a spring and a dashpot in the series).

In order to render the mechanical stress (σ)–strain (ε) curves up to the onset of yielding, the viscoelastic standard linear solid (SLS), also known as the Zener model [32], extended with an additional Maxwell element (a spring and a dashpot in the series) was applied [33] (Figure 8). It is to be noted that the single SLS model was also successfully used for polymeric systems, including polyurethanes [34,35].

The variation in σ with ε at a constant strain rate (dε/dt) can be given by Equation (11):

(11) $$\sigma =b_{1}\left[\varepsilon +b_{2}\left(1-e^{-b_{3}\varepsilon }\right)+b_{4}\left(1-e^{-b_{5}\varepsilon }\right)\right]$$

where b_1_ = E, b_2_ = (dε/dt)η_1_/E, b_3_ = E_1_/(dε/dt)/η_1_, b_4_ = (dε/dt)η_2_/E, and b_5_ = E_2_/(dε/dt)/η_2_; E, E_1_ and E_2_ are the Young’s modulus of the corresponding branches; and η_1_ and η_2_ are the viscosities of the “fluids” in dashpots 1 and 2.

**Figure 9:**
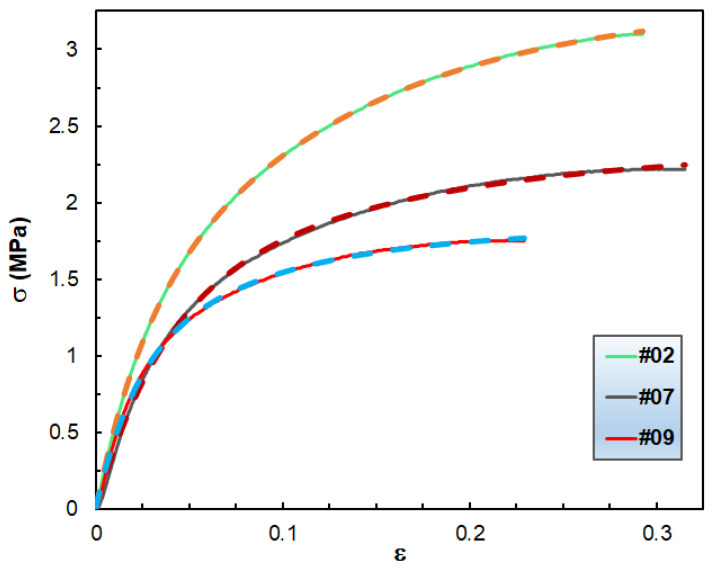
The measured (solid curves) and the fitted (dashed curves) σ versus ε data for samples #02, #07 and #09. The fitted parameters are as follows: b_1_ = 0.07 MPa, b_2_ = 14.03, b_3_ = 41.94, b_4_ = 2.31, b_5_ = 8.61 for sample #02; b_1_ = 0.02 MPa, b_2_ = 53.57, b_3_ = 29.16, b_4_ = 0.90, b_5_ = 7.52 for sample #07; and b_1_ = 0.05MPa, b_2_ = 18.20, b_3_ = 48.21, b_4_ = 1.00, b_5_ = 12.30 for sample #09. Please note that the pairs of values of (b_2_,b_3_) and (b_4_,b_5_) are interchangeable.

The derivation of Equation (11) can be found in the Supporting Information section. Equation (11) was then fitted to the experimental σ–ε data and the results of the fitting for samples #02, #07 and #09 are shown in Figure 9.

As observed in Figure 9, excellent fittings were obtained, which supports the capability of Equation (12) for rendering the σ–ε characteristics of blends of hydrocarbon wax and copolymers up to the onset of yielding. This model is also valid for the rest of the wax compositions, too, as demonstrated in the Supporting Information section (Figures S16–S28). Furthermore, it can be surmised that the additional Maxwell element, i.e., the second exponential term in Equation (12), is necessary to add because of the complexity of the studied systems. 

### 3.3. 3D Printing Application of the Blends

**Figure 10:**
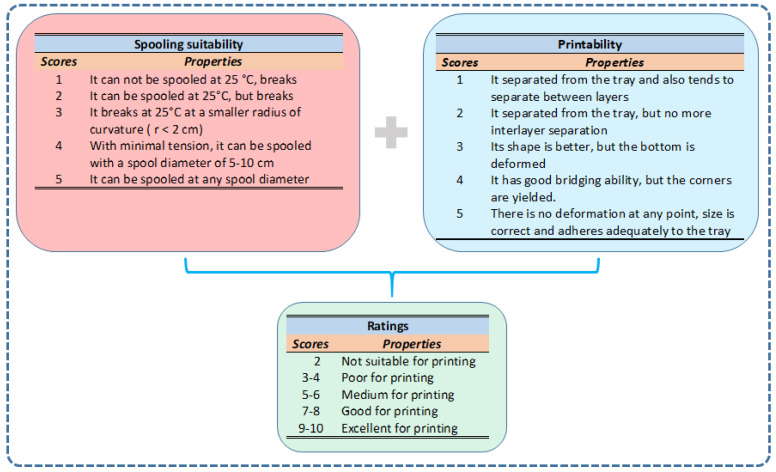
The ratings of blend samples #01–#16 according to their spooling suitability and printability.

To rate the blends in terms of 3D printabilty, we introduced two ratings: one is concerned with the spooling suitability and the other is related to the printability of the blends according to the scores detailed and compiled in Figure 10.

**Figure 11:**
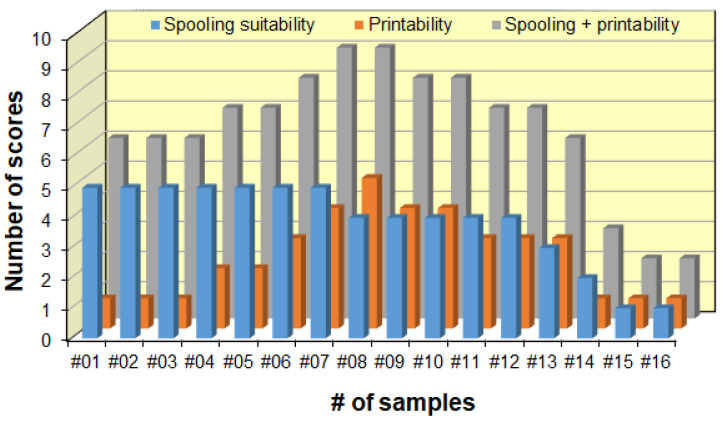
Ratings of the blends in terms of their capability for 3D printing. The scores given are detailed in Figure 10.

According to the ratings presented in Figure 10, wax samples #01–#16 were rated and the results of this process are shown in Figure 11.

**Figure 12:**
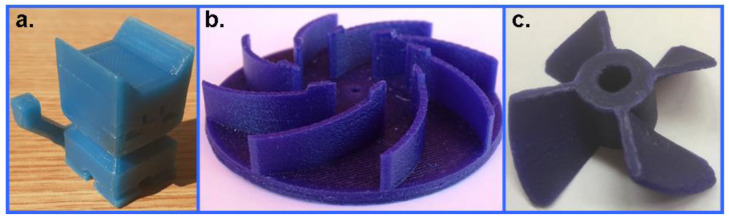
3D-printed objects printed from wax pattern #08: (**a**) Cat (small toy), (**b**) mixer and (**c**) boat propeller.

As seen in Figure 11, the spooling suitability decreases at higher Piccotex contents, i.e., lower Escorene contents. This finding can be attributed, as we have seen earlier, to the decreasing viscoelasticity with the decreasing elastic Escorene and the increasing Piccotex resin content. With a high Piccotex content, however, for samples #14–#16, corresponding to Piccotex contents betwen 65% (m/m) and 72% (m/m), the filaments tend to become very brittle. In contrast, the 3D printability is at the maximum in sample #08, while samples #07, #09 and #10 also reveal good printability. The reason behind the existence of the maximum (and its surroundings) is that with a low Piccotex content (i.e., a high Escorene content), the 3D-printed products are very soft and they cannot retain their shapes, whereas with a high Piccotex content, the viscosity at 120 °C becomes very low, and is unsuitable for printing. The resultant of the spooling suitability and the printability, i.e., the sum of their scores, forms the basis of the ratings. As it also turns out from Figure 11, the sum of the scores reveals the maxima with samples #07 and #08, corresponding to Piccotex contents of 30% (m/m) and 35% (m/m), respectively. Furthermore, as Figure 10 shows, according to the ratings, blends #07 and #08 are the most appropriate for 3D printing. Some 3D objects printed from pattern #08 are shown in Figure 12.

As it can be discerned from Figure 12, the wax pattern is indeed suitable for the production of 3D wax objects for investment casting. 

## 4. Conclusions

The rheological and mechanical properties of three-component wax blends were thoroughly studied. The wax patterns that were intended to be used for 3D printing consisted of a microcrystalline hydrocarbon wax as a base component, an ethylene–vinyl acetate copolymer (Escorene) for improving the flexibility and a poly (α–methyl stryrene–co–vinyl toluene) copolymer (Piccotex 75) for increasing the shape fixity. However, in the preliminary experiments, it was established that the hydrocarbon wax content in these blends should not exceed 30% (m/m) in order to obtain patterns with acceptable consistency. Therefore, the wax content was kept constant at 28% (m/m), while the Piccotex and Escorene contents were varied simultaneously in the range from 0% (m/m) to 72% (m/m). The rheological properties of the blends as a function of the composition and temperature were interpreted in terms of a stretched exponential model and the Arrhenius–Guzman equation, respectively. The use of a stretched exponential function for the description of the variation in the viscosities with the composition may also be applied in the case of other two- or three-component blends. It was found that near the pour point, the shear stress increased with the shear rate as expected; however, at higher rates, the shear stress started to decrease. This finding was attributed to the shear-stress-induced crystallization. For rendering this behavior, the Houska model was successfully employed. The yield stress was observed to change linearly with the Piccotex content, simplifying the engineering. However, the Young’s modulus was found to vary according to a sigmoid curve. The viscoelastic properties of the wax blends were described by means of the SLS model containing two Maxwell elements. It is to be noted that the SLS model can generally be applied for the constitutive modeling of the viscoelastic properties of various polymeric systems. The blends with various Piccotex and Escorene contents were qualified for 3D printing in terms of spooling suitability and printability. According to the ratings, the spooling suitability decreased, whereas the printability had a maximum with the Piccotex content. By taking into account both the spooling suitability and printability, it was found that the most suitable patterns for 3D printing were those that contained 30% (m/m) and 35% (m/m) Piccotex. The properties of the wax blends can be further improved and tailored by using hydrocarbon waxes with a higher pour point (e.g., DMW 8595) and/or applying fillers. 

## Figures and Tables

**Table 1:** The compositions of the wax samples made of microcrystalline wax (DMW7478), Piccotex 75 and Escorene.

Sample	DMW7478 % (m/m)	Piccotex 75 % (m/m)	Escorene % (m/m)
#01	28	0	72
#02	28	5	67
#03	28	10	62
#04	28	15	57
#05	28	20	52
#06	28	25	47
#07	28	30	42
#08	28	35	37
#09	28	40	32
#10	28	45	27
#11	28	50	22
#12	28	55	17
#13	28	60	12
#14	28	65	7
#15	28	70	2
#16	28	72	0

**Table 2:** Pour points for samples #01–#16.

**Sample**	**#01**	**#02**	**#03**	**#04**	**#05**	**#06**	**#07**	**#08**
Pour point (°C)	72.8	–	–	72.2	71.9	72.0	71.5	70.6
**Sample**	**#09**	**#10**	**#11**	**#12**	**#13**	**#14**	**#15**	**#16**
Pour point (°C)	71.3	71.2	70.8	70.6	69.5	70.7	71.1	72.0

**Table 3:** The Young’s modulus, yield stress, yield strain, stress at break, strain at break and flexural modulus for the wax samples.

Sample	Young’s Modulus (MPa)	Yield Stress (MPa)	Yield Strain %	Stress at Break (MPa)	Strain at Break (%)	Flexural Modulus (MPa)
#01	58.24 ± 6.99	3.51 ± 0.11	27.5 ± 2.1	3.08 ± 0.16	29.9 ± 2.4	62.50 ± 1.46
#02	52.63 ± 2.18	3.12 ± 0.06	29.2 ± 1.8	2.80 ± 0.08	32.7 ± 2.0	57.10 ± 1.51
#03	52.57 ± 3.54	3.06 ± 0.06	28.9 ± 0.8	2.76 ± 0.07	31.7 ± 1.4	50.00 ± 1.93
#04	45.01 ± 5.10	2.75 ± 0.04	29.4 ± 1.6	2.43 ± 0.12	34.6 ± 0.9	54.10 ± 2.31
#05	45.40 ± 4.91	1.35 ± 0.42	6.3 ± 3.2	0.95 ± 0.51	9.6 ± 3.2	51.52 ± 1.76
#06	51.60 ± 4.22	2.42 ± 0.07	24.3 ± 1.3	1.75 ± 0.38	26.3 ± 1.6	45.81 ± 3.39
#07	48.41 ± 6.74	2.16 ± 0.02	28.0 ± 0.8	1.68 ± 0.22	37.6 ± 1.4	50.63 ± 2.98
#08	60.30 ± 3.44	2.16 ± 0.04	22.5 ± 0.5	1.17 ± 0.05	27.6 ± 2.1	51.53 ± 0.59
#09	55.09 ± 4.67	1.83 ± 0.07	21.1 ± 1.8	1.09 ± 0.07	30.7 ± 3.6	55.06 ± 3.73
#10	49.90 ± 4.47	1.63 ± 0.01	18.8 ± 1.3	1.10 ± 0.05	37.8 ± 1.0	58.35 ± 2.52
#11	64.16 ± 4.36	1.61 ± 0.01	18.0 ± 0.8	1.06 ± 0.03	36.9 ± 1.8	55.91 ± 3.69
#12	64.10 ± 7.32	1.57 ± 0.06	12.8 ± 1.7	1.00 ± 0.10	21.3 ± 5.2	70.95 ± 6.57
#13	85.67 ± 11.50	1.58 ± 0.05	10.9 ± 0.25	0.98 ± 0.07	25.6 ± 6.5	70.34 ± 3.12
#14	99.50 ± 17.68	1.78 ± 0.03	11.9 ± 0.6	1.05 ± 0.05	40.6 ± 7.3	74.62 ± 4.65
#15	116.22 ± 21.46	1.37 ± 0.14	5.9 ± 0.6	1.18 ± 0.27	11.5 ± 3.6	128.29 ± 7.94
#16	108.50 ± 12.10	1.11 ± 0.05	6.3 ± 0.3	0.37 ± 0.06	31.4 ± 8.5	200.48 ± 27.63

## Data Availability

The data used for the research are available upon request.

## Supplementary Materials

The following supporting information can be downloaded at: https://www.mdpi.com/article/10.3390/polym14214744/s1, Figure S1: The GC-FID traces of DMW7478 wax; Figure S2: MALDI-TOF MS spectrum of Piccotex75; Figure S3: Experimental shear stress versus shear rate curves for samples #01, #02 and #03 recorded at 90 °C; Figure S4: Experimental shear stress versus shear rate curves for samples #04, #05 and #06 recorded at 90 °C; Figure S5: Experimental shear stress versus shear rate curves for samples #08, #09 and #10 recorded at 90 °C; Figure S6: Experimental shear stress versus shear rate curves for samples #11, #12 and #13 recorded at 90 °C; Figure S7: Experimental shear stress versus shear rate curves for samples #14, #15 and #16 recorded at 90 °C; Figure S8: Experimental shear stress versus shear rate curves for sample #16 recorded at 75 °C; Figure S9: Viscosity versus shear rate curve for sample #07 recorded at 75 °C; Figure S10: Viscosity versus shear rate curve for sample #07 recorded at 80 °C; Figure S11: Viscosity versus shear rate curve for sample #07 recorded at 85 °C; Figure S12: Viscosity versus shear rate curve for sample #07 recorded at 90 °C; Figure S13: Viscosity versus shear rate curve for sample #07 recorded at 100 °C; Figure S14: Viscosity versus shear rate curve for sample #07 recorded at 110 °C; Figure S15: Images for sample #07 at shear rates 30 s^−1^ (a) and 100 s^−1^ (b) at temperature of 85 °C; Figure S16: The measured and the fitted σ versus ε data for the sample #01; Figure S17: The measured and the fitted σ versus ε data for the sample #03; Figure S18: The measured and the fitted σ versus ε data for the sample #04; Figure S19: The measured and the fitted σ versus ε data for the sample #05; Figure S20: The measured and the fitted σ versus ε data for the sample #06; Figure S21: The measured and the fitted σ versus ε data for the sample #08; Figure S22: The measured and the fitted σ versus ε data for the sample #10; Figure S23: The measured and the fitted σ versus ε data for the sample #11; Figure S24: The measured and the fitted σ versus ε data for the sample #12; Figure S25: The measured and the fitted σ versus ε data for the sample #13; Figure S26: The measured and the fitted σ versus ε data for the sample #14; Figure S27: The measured and the fitted σ versus ε data for the sample #15; Figure S28: The measured and the fitted σ versus ε data for the sample #16; Table S1: The values of A and B of the Arrhenius-Guzman equation determined by fitting.

## References

[1] Singh S., Singh R. (2017). Precision investment casting: A state of art review and future trends. J. Eng. Manuf..

[2] Pattnaik S., Karunakar D.B., Jha P.K. (2012). Developments in investment casting process—A review. J. Mater. Process. Technol..

[3] Tulloch A.P. (1980). Beeswax—Composition and Analysis. Bee World.

[4] Jones S., Yuan C. (2003). Advances in shell moulding for investment casting. J. Mater Process. Technol..

[5] Cheah C.M., Chua C.K., Lee C.W., Feng C., Totong K. (2005). Rapid prototyping and tooling techniques: A review of applications for rapid investment casting. Int. J. Adv. Manuf. Technol..

[6] Chakravorty S. (1999). The Properties of Waxes Used in the Investment Casting Industry: Final Report.

[7] Tewo R.K., Rutto H.L., Focke W., Seodigeng T., Koech L.K. (2019). Formulations, development and characterization techniques of investment casting patterns. Rev. Chem. Eng..

[8] Muschio H.M. (1995). Filler and Wax Composition for Investment Casting. U.S. Patent.

[9] Bemblage O., Karunakar D.B. A study on the blended wax patterns in investment casting process. Proceedings of the World Congress on Engineering.

[10] Zhou L.Y., Fu J.Z., He Y. (2020). A Review of 3D Printing Technologies for Soft Polymer Materials. Adv. Func. Mater..

[11] Kiefer L.A., Lee Y.J. (2021). Three–Dimensional Printable Wax Material for Casting Comprises Carnauba Wax and Polymer. U.S. Patent.

[12] Mukhtarkhanov M., Shehab E., Ali M.H. (2022). Process Parameter Optimization for 3D Printed Investment Casting Wax Pattern and Its Post–Processing Technique. Appl. Sci..

[13] Fan Z., Tang C., Fan D. (2018). Laser Sintering 3D Printing Precision Casting Wax Powder Material Useful in 3D Printing, Comprises Petroleum Wax, Beeswax, Lignite, Polyethylene Wax, Resin, Toughening Agent, Stearic Acid, Flatting Agent, and Surfactant. Chinese Patent.

[14] Ke Y., Ye S., Yu C., Zhao W., Pan Y., Guo X. (2017). Industrial Casting Three–Dimensional Wax Printing Wire Material Comprises Industrial Wax, Polyolefin Plastomer Resin, Polyethylene Wax, and Stearic Acid. Chinese Patent.

[15] Kuo C.C., Chen W.H., Li J.F., Zhu Y.J. (2018). Development of a flexible modeling base for additive manufacturing. Int. J. Adv. Manuf. Technol..

[16] Lan Z., Huang J., Li D. (2021). Three–Dimensional Printing Wax Material Useful for Jewelry Comprises Petroleum Wax, Animal Wax, Vegetable Wax, Mineral Wax, Synthetic Wax, Viscosity Regulator, Toughening Agent, Hardness Regulator, Sodium Starch Octenyl Succinate and Toner. Chinese Patent.

[17] Srinivasan D., Meignanamoorthy M., Ravichandran M., Mohanavel V., Alagarsamy S.V., Chanakyan C., Sakthivelu S., Karthick A., Prabhu T.R., Rajkumar S. (2022). 3D Printing Manufacturing Techniques, Materials, and Applications: An Overview. Adv. Mater. Sci. Eng..

[18] Mezger T.G. (2011). The Rheology Handbook.

[19] Roschochowski A., Matuszak A. (2000). Rapid tooling: The state of the art. J. Mater. Process. Technol..

[20] Oliveira L.M.S.L., Nunes R.C.P., Pessoa L.M.B., Reis L.G., Spinelli L.S., Lucas E.F. (2020). Influence of the chemical structure of additives poly(ethylene–co–vinyl acetate)–based on the pour point of crude oils. J. Appl. Polym. Sci..

[21] Kolczyk J., Jamrozowicz Ł., Kaźnica N. (2017). Rheological Properties of Typical Ceramic Slurries Used in the Lost Wax Technology. Archi. Foundry Eng..

[22] Widemann M., Driest P.J., Orecchia P., Naline F., Golling F.E., Hecking A., Eggert C., Pires R., Danielmeier K., Richter F.U. (2018). Structure–Property Relations in Oligomers of Linear Aliphatic Diisocyanates. ACS Sustain. Chem. Eng..

[23] Spann A.P., Hancock M.J., Rostami A.A., Platt S.P., Rusyniak M.J., Sundar R.S., Lau R.W., Pithawalla Y.B. (2021). Viscosity Model for Liquid Mixtures of Propylene Glycol, Glycerol, and Water. Ind. Eng. Chem. Res..

[24] Mendes R., Vinay G., Ovarlez G., Coussot P. (2015). Modeling the rheological behavior of waxy crude oils as a function of flow and temperature history. J. Rheol..

[25] Shan L., Tan Y., Kim Y.R. (2012). Applicability of the Cox–Merz relationship for asphalt binder. Constr. Build. Mater..

[26] Ji X., Liang Y., Cao W. (2022). Effect of Solid Volume Concentration on Rheological Properties of Chengdu Clay Slurry. Processes.

[27] Sestak J., Charles M.E., Cawkwell M.G., Houska M. (1987). Start–up of gelled crude oil pipelines. J. Pipelines.

[28] Cawkwell M.G., Charles M.E. (1989). Characterization of Canadian artic thixotropic gelled crude oils utilizing an eight–parameter model. J. Pipelines.

[29] Ashbaugh H.S., Guo X., Schwahn D., Prud’homme R.K., Richter D., Fetters L.J. (2005). Interaction of Paraffin Wax Gels with Ethylene/Vinyl Acetate Co–polymers. Energy Fuels.

[30] Li N., Mao G., Shi X., Tian S., Liu Y. (2018). Advances in the research of polymeric pour point depressant for waxy crude oil. J. Dispers. Sci. Technol..

[31] Zhang H., Liu N., Ran X., Han C., Han L., Zhuang Y., Dong L. (2012). Toughening of Polylactide by Melt Blending with Methyl Methacrylate–Butadiene–Styrene Copolymer. J. Appl. Polym. Sci..

[32] Ward I.M., Sweeney J. (2004). An Introduction to the Mechanical Properties of Solid Polymers.

[33] Karger–Kocsis J., Kéki S. (2018). Review of Progress in Shape Memory Epoxies and Their Composites. Polymers.

[34] Nagy L., Nagy M., Vadkerti B., Daróczi L., Deák G., Zsuga M., Kéki S. (2019). Designed Polyurethanes for Potential Biomedical and Pharmaceutical Applications: Novel Synthetic Strategy for Preparing Sucrose Containing Biocompatible and Biodegradable Polyurethane Networks. Polymers.

[35] Kordován M.A., Hegedűs C., Czifrák K., Lakatos C., Kálmán-Szabó I., Daróczi L., Zsuga M., Kéki S. (2022). Novel Polyurethane Scaffolds Containing Sucrose Crosslinker for Dental Application. Int. J. Mol. Sci..

