# Supplementary material for: Flexible Investment Casting Wax Patterns for 3D-Printing: Their Rheological and Mechanical Characterizations
Source: Polymers (Basel). 2022 Nov 5;14(21):4744. doi: 10.3390/polym14214744 (PMC9657807; doi:10.3390/polym14214744)
Supplement: Supplementary file 1 [file polymers-14-04744-s001.zip › polymers-1955392-supplementary.pdf]

Supporting Information for

Flexible investment casting wax patterns for 3D-printing: their rheological and mechanical characterizations

László Szabó[1,2], György Deák[3], Dávid Nyúl[4], Sándor Kéki[3*]

[1]Bogdány Petrol Ltd.,, Gyártelep, H-4511 Nyírbogdány, Hungary

[2]Doctoral School of Chemistry, University of Debrecen, Egyetem tér 1, H-4032 Debrecen, Hungary

[3]Department of Applied Chemistry, Faculty of Sciences and Technology, University of Debrecen, Egyetem tér 1, H-4032 Debrecen, Hungary

[4]Department of Physical Chemistry, Faculty of Sciences and Technology, University of Debrecen, Egyetem tér 1, H-4032 Debrecen, Hungary

Table of Contents

1. Gas-chromatographic (GC) analysis of DMW7478 wax (Figure S1) 2

2. Matrix-Assisted Laser Desorption/Ionization Time-of-Flight Mass Spectrometry (MALDI-TOF MS) (Figure S2) 3

3. Temperature dependence of the viscosity (Table S1) 4

4. Dependence of the shear stress on the shear rate (Figure S3-S8) 5

5. Derivation of eq. 12. 12

6. Dependence of the tensile stress on the relative strain (Figure S16-S28)...........................14

1. Gas-chromatographic (GC) analysis of DMW7478 wax (Figure S1)

The gas-chromatographic (GC) measurements were performed by a Shimadzu 2030 type gas-chromatograph. Sample was dissolved in cyclohexane at a concentration of 0.5 mg/mL and 1 μL was injected into the chromatograph. The following experimental parameters were used:

Injector: 290°C, Injection Mode: splitless, Flow Control Mode: Linear velocity, Carrier Gas: Helium, Pressure: 80 kPa, Total Flow: 50 mL/min, Column Flow: 2,55 mL/min, Linear Velocity: 40 cm/s, Purge Flow: 3 mL/min, Column: ZB-1HT Inferno, Lenght: 30.0 m, Inner Diameter: 0.32 mm, Film Thickness: 0.1 μm, Total Program Time: 49 min, Detector: FID, Detector temperature: 390°C, Column Oven Temperature Program: Temperature: 50 °C (hold time: 2 min), heating rate: 8 °C/min up to 390 °C (hold time: 4.5 min).

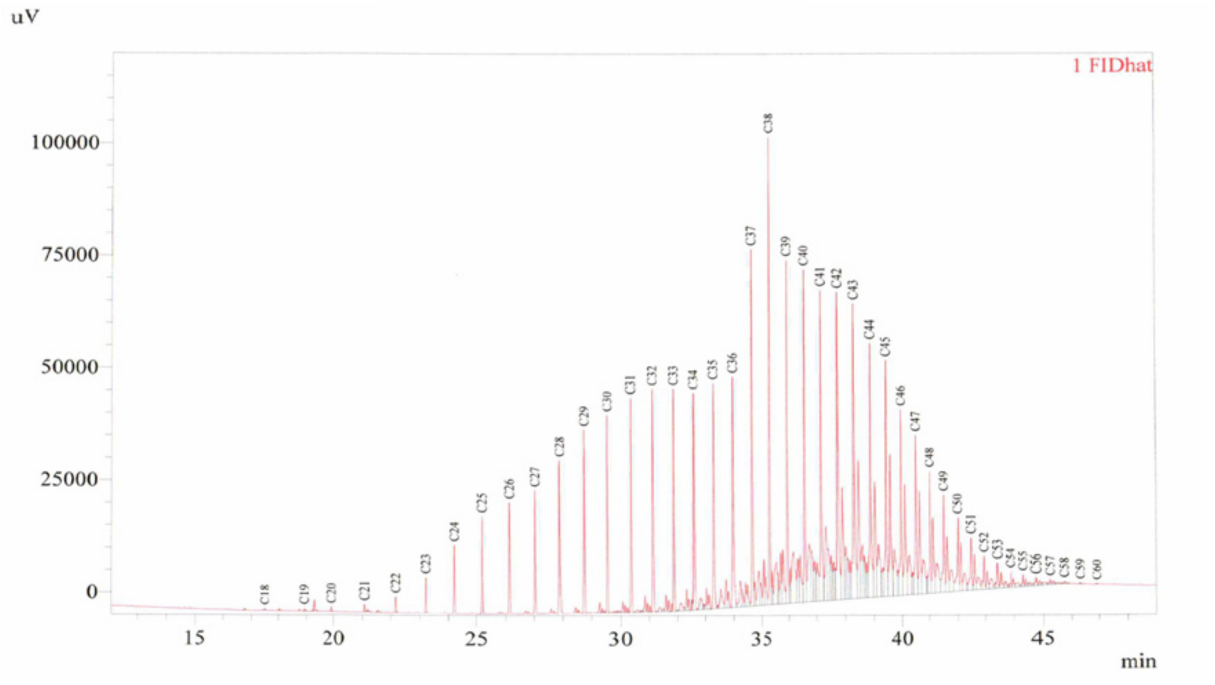

Figure S1. The GC-FID traces of DMW7478 wax.

2. Matrix-Assisted Laser Desorption/Ionization Time-of-Flight Mass Spectrometry (MALDI-TOF MS) (Figure S2)

The MALDI-TOF MS measurements were carried out with a Bruker Autoflex Speed mass spectrometer (Bruker Daltonik, Bremen, Germany). Reflectron mode was used for all of the measurements where 19 kV (ion source voltage 1), 16.65 kV (ion source voltage 2), 21 kV (reflector voltage 1) and 9.55 kV (reflector voltage 2) were applied. The solid phase laser (355 nm) was operated at 200 Hz and 2000 shoots were summed. The spectra were externally calibrated with polyethylene glycol (M_n = 1540 g/mol). The samples were prepared with dithranol (1,8-dihydroxy-9-anthrone) dissolved in tetrahydrofuran at a concentration of 20 mg/mL, the Piccotex was also solved in tetrahydrofuran (concentration 10 mg/mL). Silver tri-fluoroacetate solution were used as ionizing agent at a concentration of 5 mg/mL in tetrahydrofuran. The solutions of the matrix, sample and the ionizing agent were mixed in the ratio of 5:2:1, respectively.

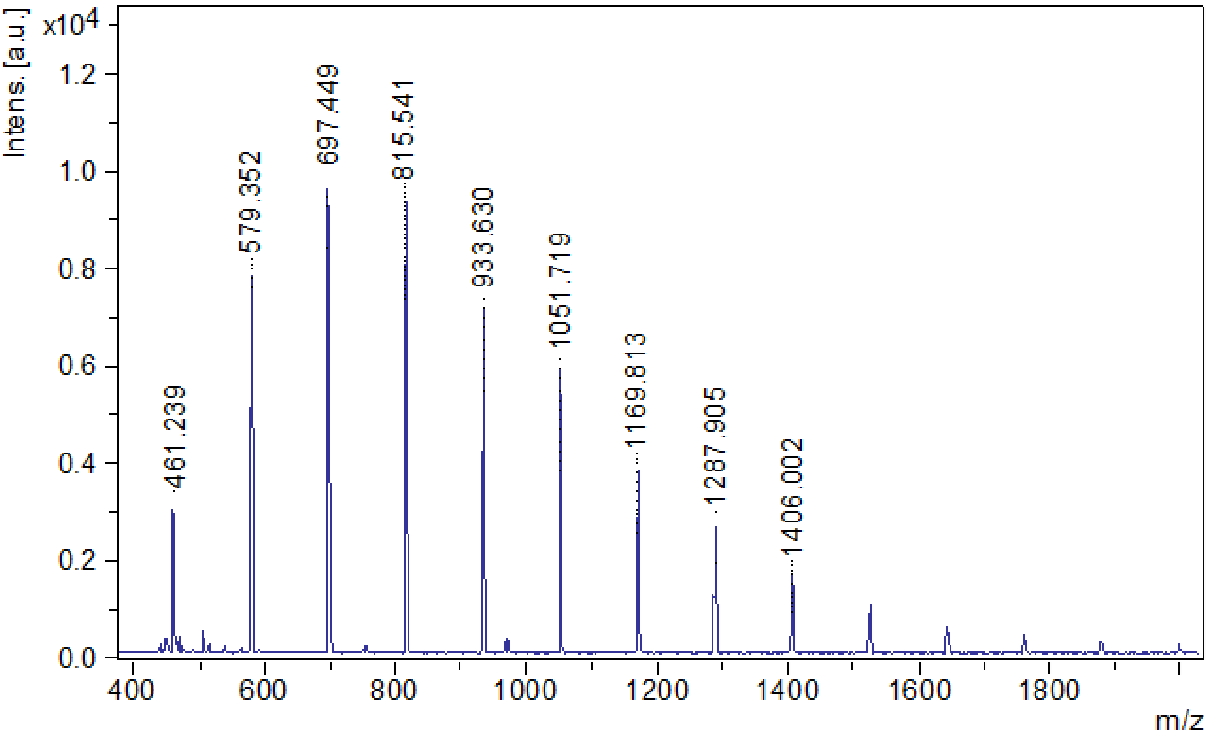

Figure S2. MALDI-TOF MS spectrum of Piccotex75. The copolymer was cationized with silver ions, i.e., $[M+Ag]^+$ adduct ions were formed. The numbers shown on the top of peaks are the m/z value of $[M+^{107}Ag]^+$. The mass difference between the adjacent peaks corresponds to the mass of α-methyl-styrene or the isomer vinyl toluene unit (118.09).

3. Temperature dependence of the viscosity (Table S1)

Table S1. The values of A and B of the Arrhenius-Guzman equation determined by fitting.

Sample	$A*10^8$	B (K)
#01	2176	5469
#02	1651	5498
#03	934	5602
#04	630	5655
#05	440	5685
#06	230	5828
#07	140	5865
#08	66	6023
#09	41	6062
#10	18	6241
#11	7.8	6359
#12	2.5	6614
#13	0.78	6856
#14	0.048	7706
#15	0.0014	8787
#16	-	-

4. Dependence of the shear stress on the shear rate (Figure S3-S8)

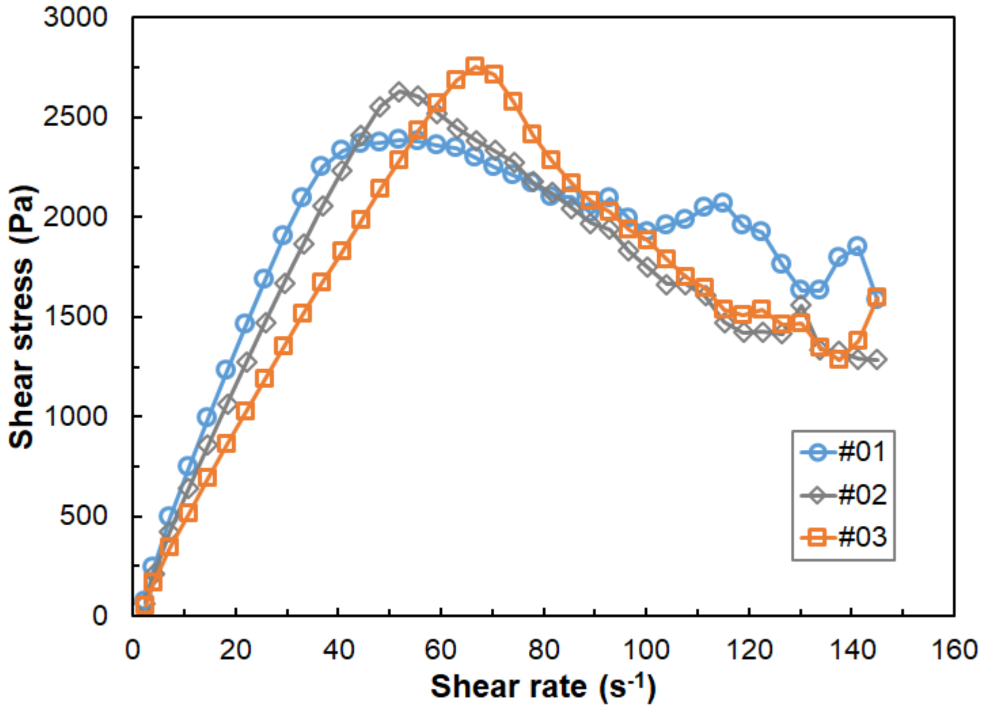

Figure S3. Experimental shear stress *versus* shear rate curves for samples #01, #02 and #03 recorded at 90 °C.

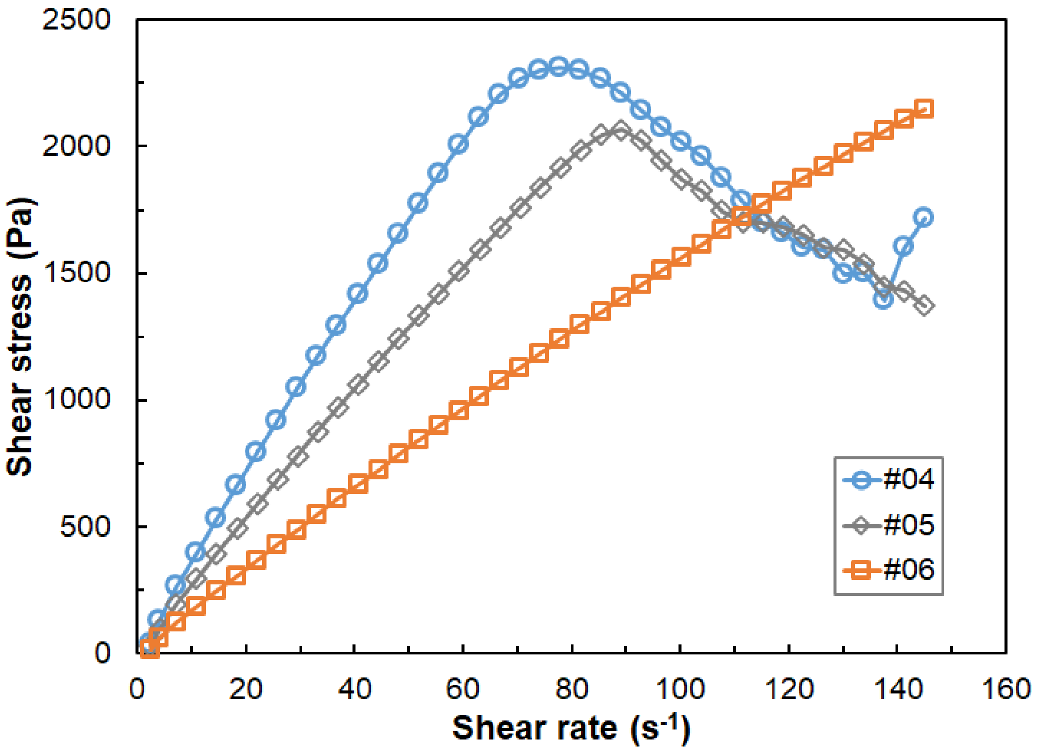

Figure S4. Experimental shear stress *versus* shear rate curves for samples #04, #05 and #06 recorded at 90 °C.

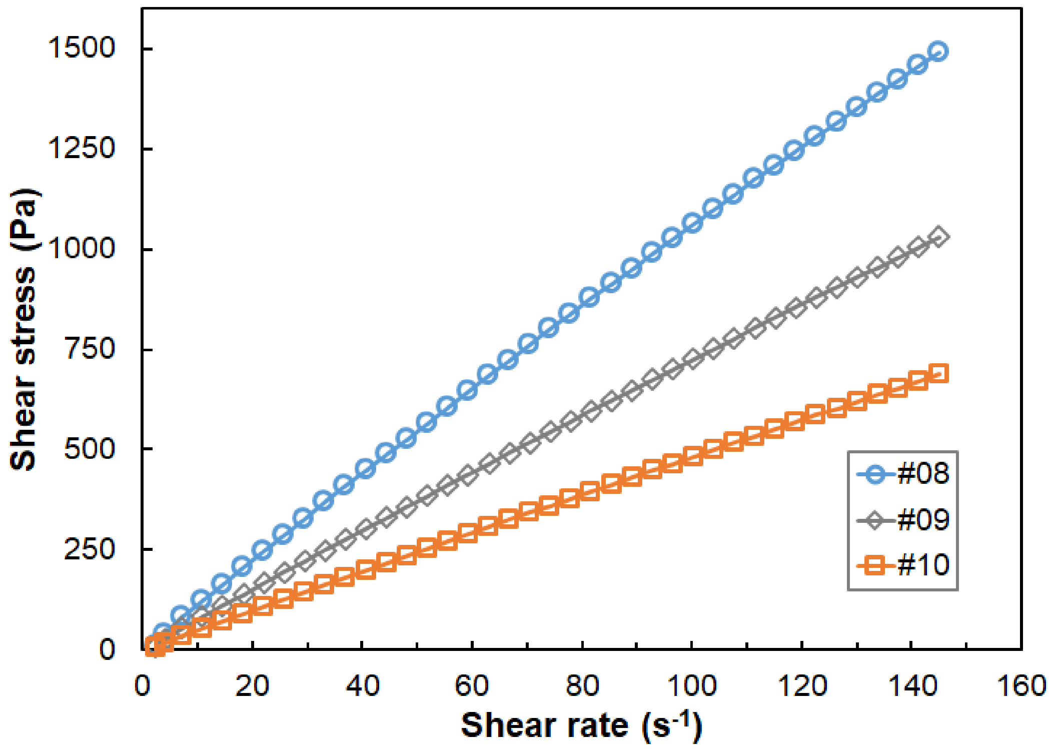

Figure S5. Experimental shear stress *versus* shear rate curves for samples #08, #09 and #10 recorded at 90 °C. (sample #07 data are plotted in Figure 5)

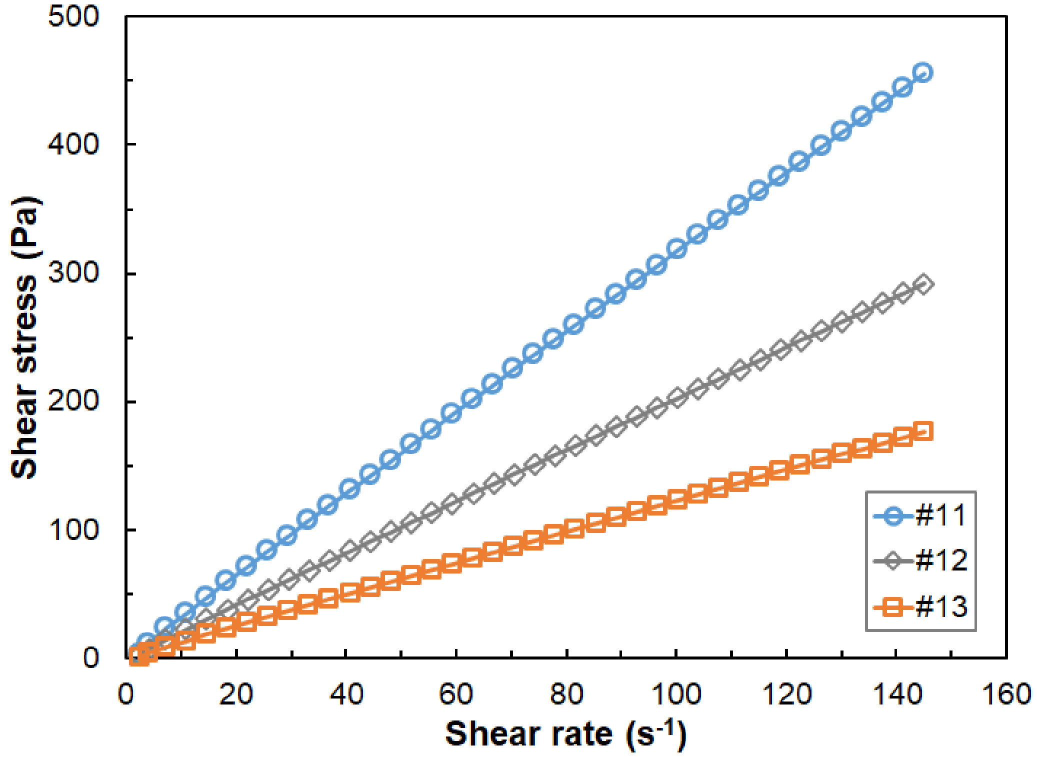

Figure S6. Experimental shear stress *versus* shear rate curves for samples #11, #12 and #13 recorded at 90 °C.

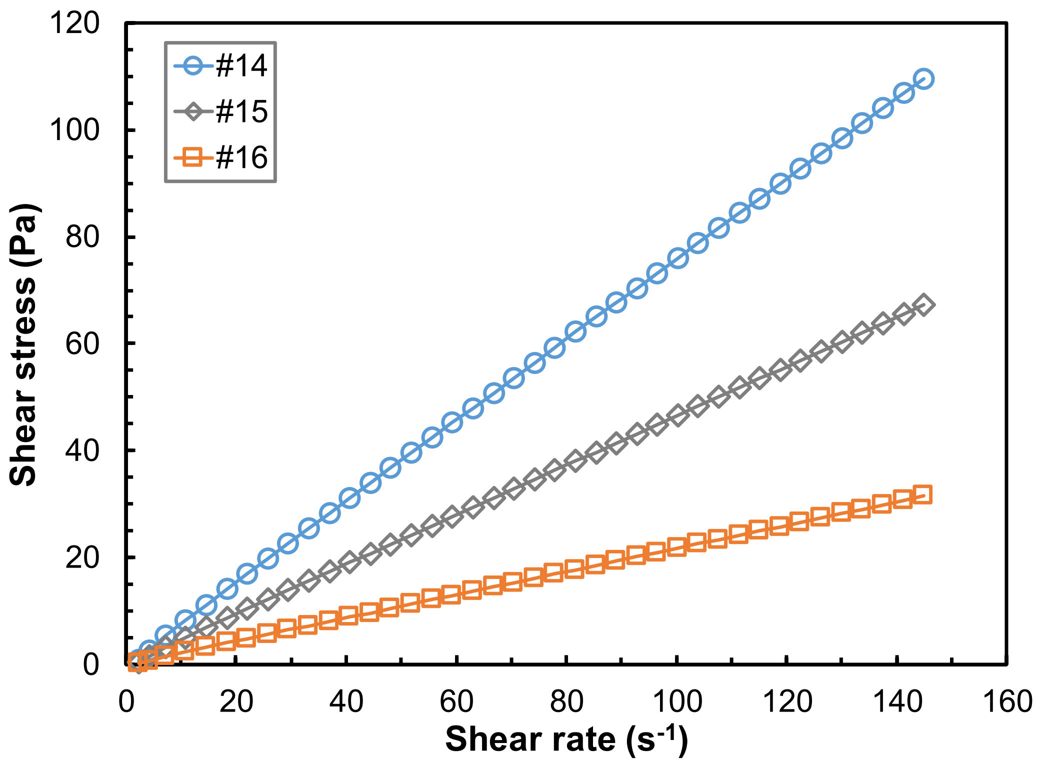

Figure S7. Experimental shear stress *versus* shear rate curves for samples #14, #15 and #16 recorded at 90 °C.

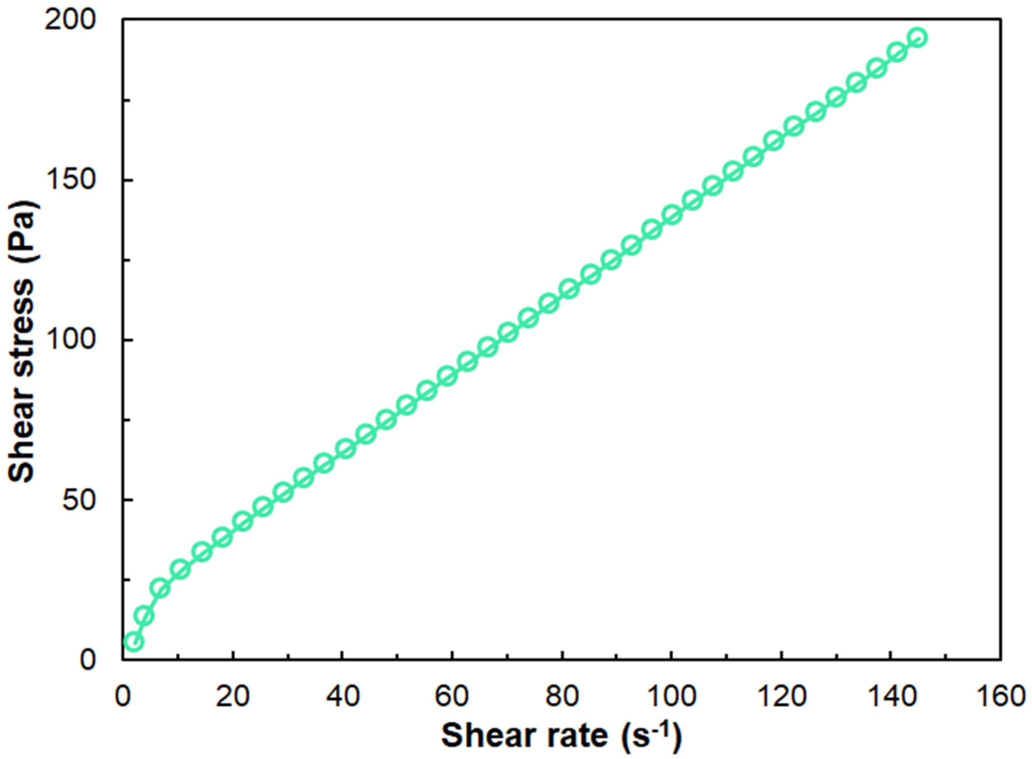

Figure S8. Experimental shear stress *versus* shear rate curves for sample #16 recorded at 75 °C.

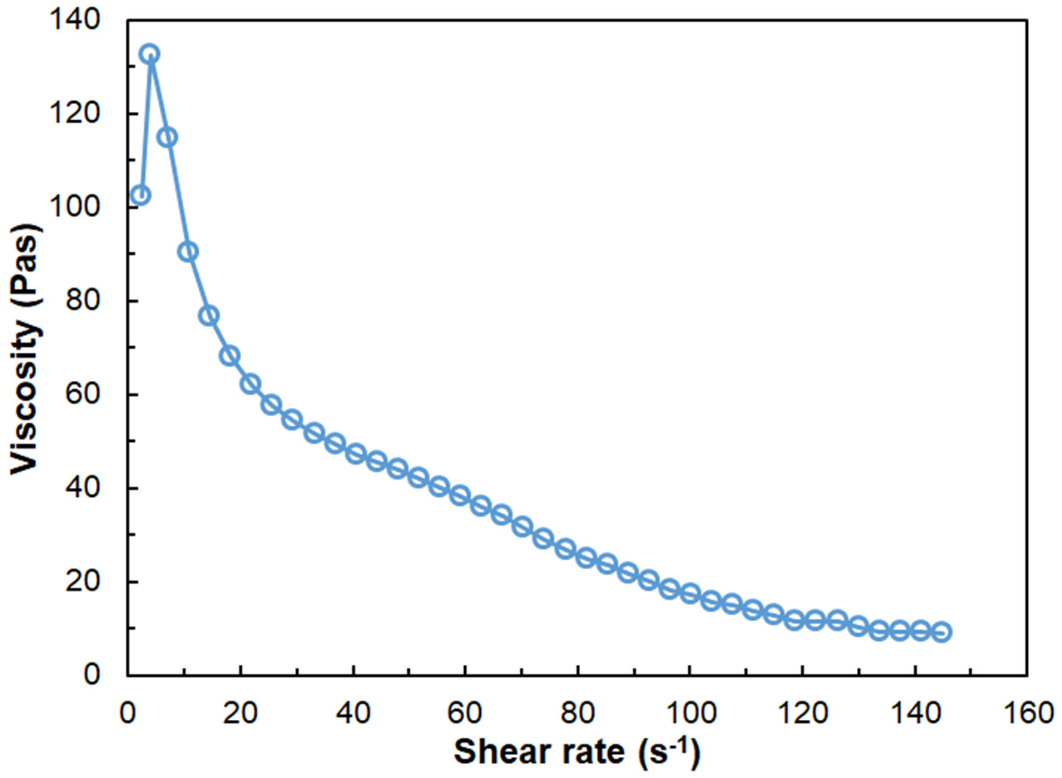

Figure S9. Viscosity *versus* shear rate curve for sample #07 recorded at 75 °C.

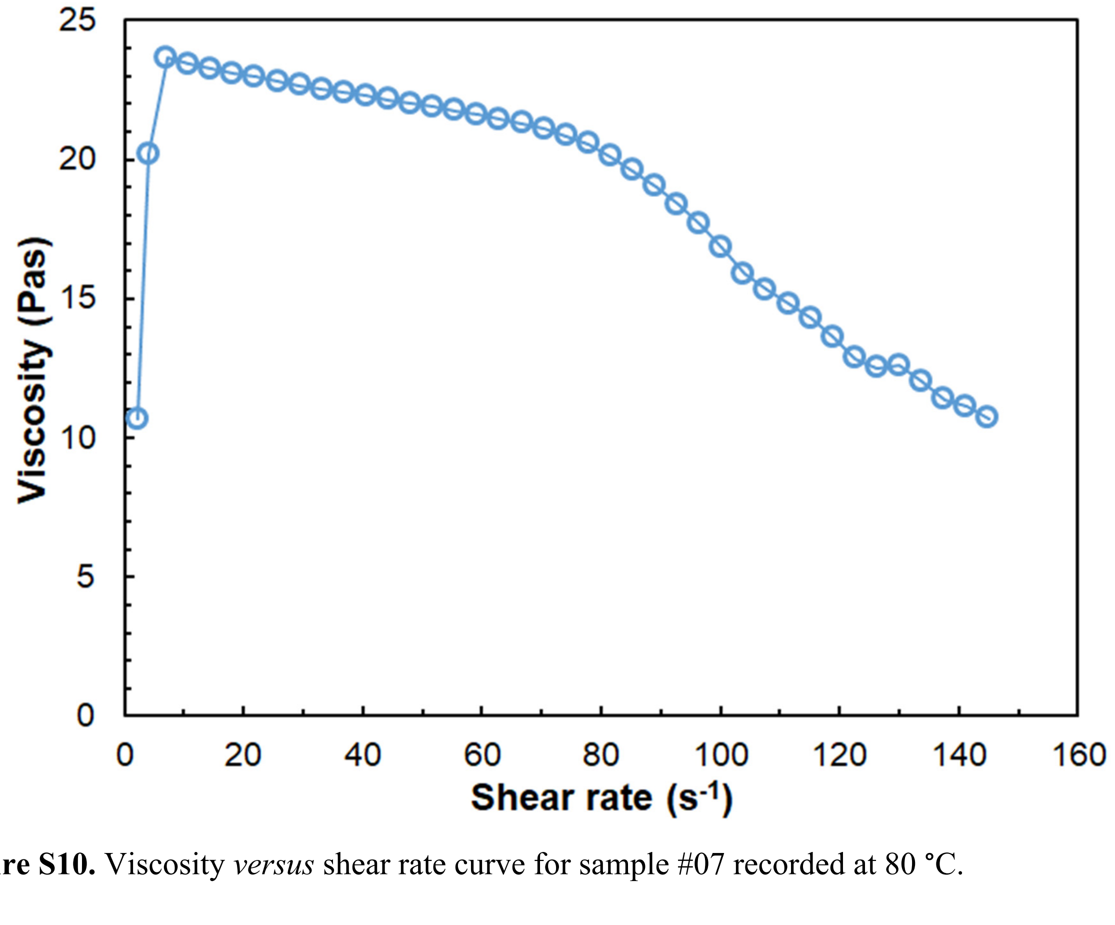

Figure S10. Viscosity *versus* shear rate curve for sample #07 recorded at 80 °C.

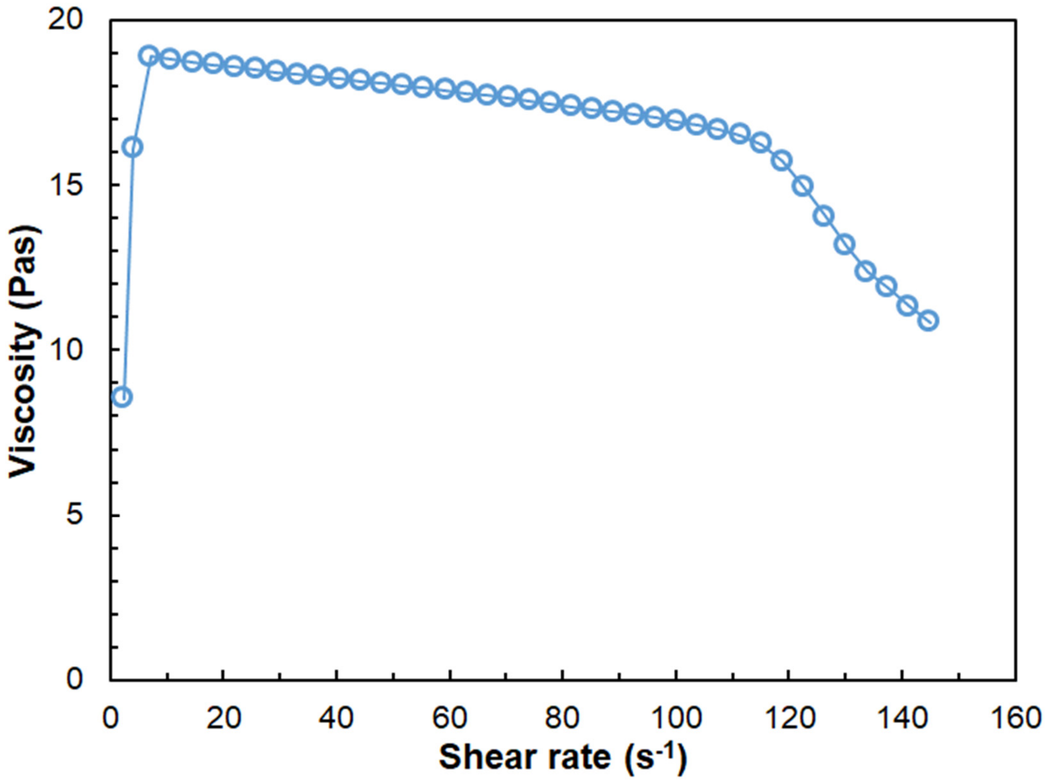

Figure S11. Viscosity *versus* shear rate curve for sample #07 recorded at 85 °C.

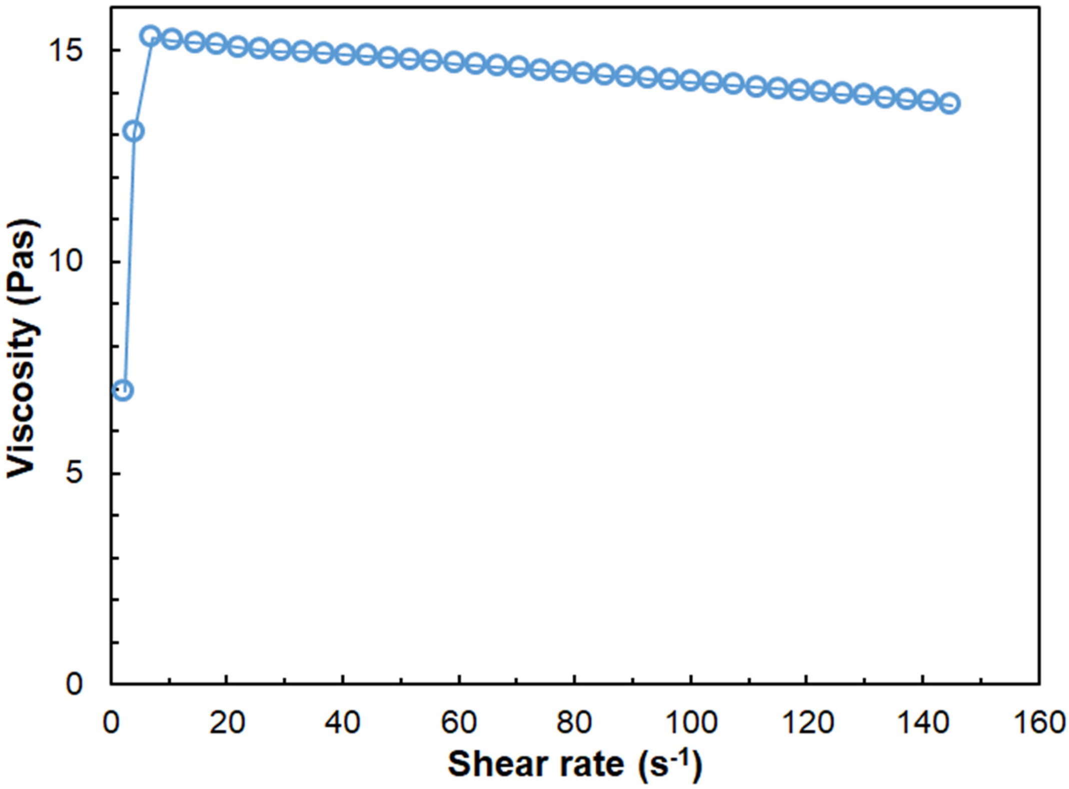

Figure S12. Viscosity *versus* shear rate curve for sample #07 recorded at 90 °C.

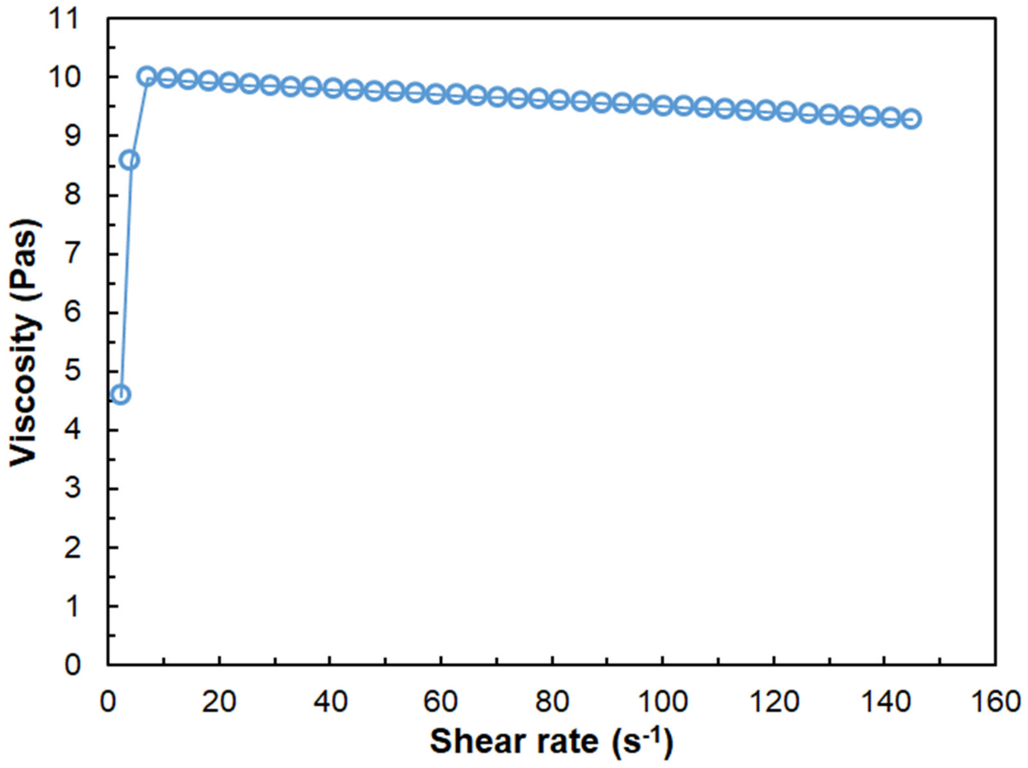

Figure S13. Viscosity *versus* shear rate curve for sample #07 recorded at 100 °C.

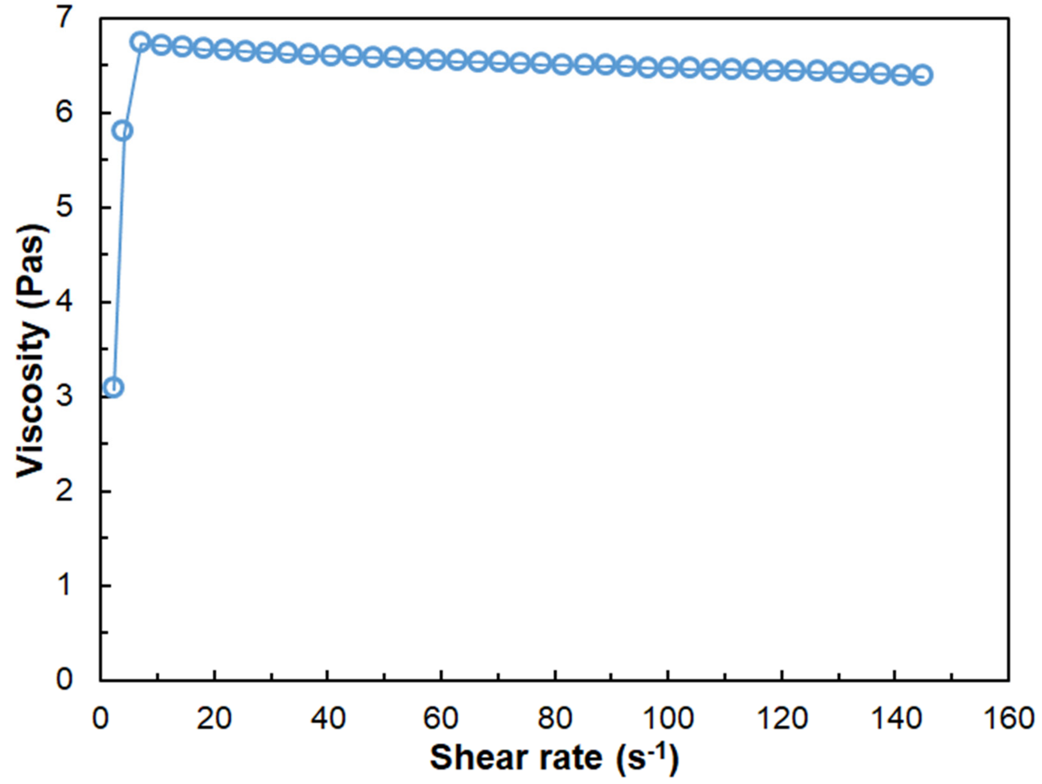

Figure S14. Viscosity *versus* shear rate curve for sample #07 recorded at 110 °C.

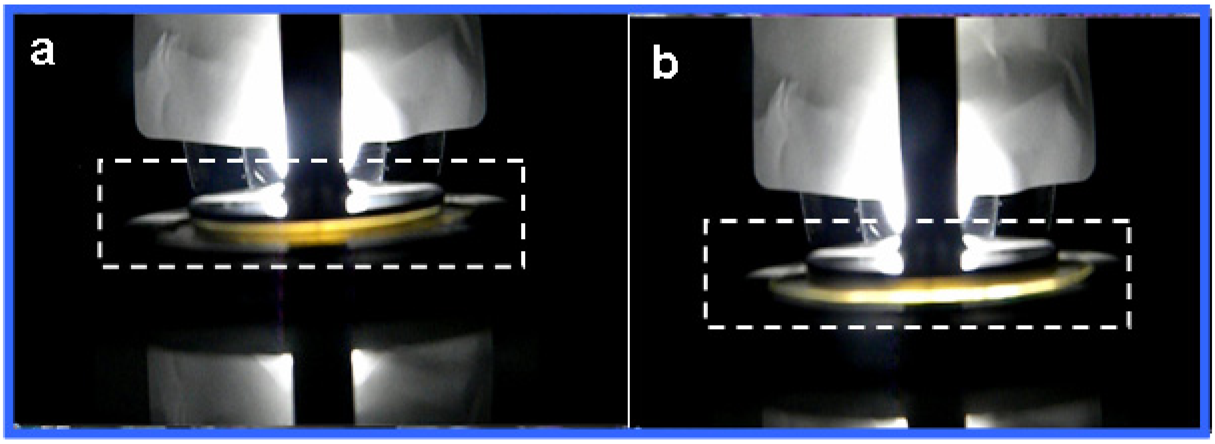

Figure S15. Images for sample #07 at shear rates 30 s^{-1} (a) and 100 s^{-1} (b) at temperature of 85 °C. The dashed line area denotes the thin liquid layer.

5. Derivation of eq. 12.

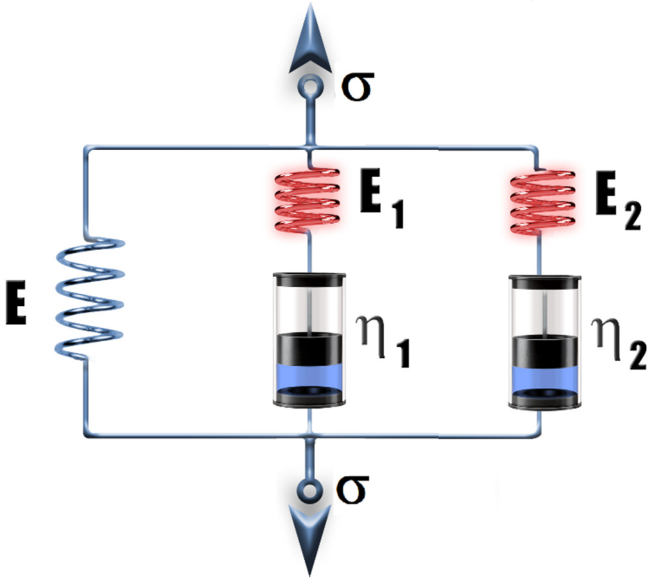

According to the above SLS model with two Maxwell elements, the following relationships can be established for the stresses (σ) and strains (ε):

$$\sigma = E\varepsilon + E_1\varepsilon_1 + E_2\varepsilon_2 \tag{S1}$$

$$\varepsilon = \varepsilon_1 + \varepsilon'_1 = \varepsilon_2 + \varepsilon'_2 \tag{S2}$$

$$\frac{d\varepsilon}{dt} = \frac{d\varepsilon_1}{dt} + \frac{d\varepsilon'_1}{dt} \tag{S3}$$

$$\frac{d\varepsilon}{dt} = \frac{d\varepsilon_2}{dt} + \frac{d\varepsilon'_2}{dt} \tag{S4}$$

$$E_1\varepsilon_1 = \eta_1 \frac{d\varepsilon'_1}{dt} \tag{S5}$$

$$E_2\varepsilon_2 = \eta_2 \frac{d\varepsilon'_2}{dt} \tag{S6}$$

where E, E_1 and E_2 are the Young`s modulus of the corresponding branches, and η_1 and η_2 are the viscosities of the "fluids" in the dashpot 1 and 2. ε_1,ε_2 and ε_1',ε_2' are the strain (relative) for the springs and the dashpots, respectively.

Expressing $\frac{d\varepsilon'_1}{dt}$ from eq. S5 and $\frac{d\varepsilon'_2}{dt}$ from eq. S6 and substituting them into eq. S3 and eq. S4, eq. S7 and eq. S8 come as:

$$\frac{d\varepsilon}{dt} = \frac{d\varepsilon_1}{dt} + \frac{E_1}{\eta_1}\varepsilon_1 \qquad \text{(S7)}$$

$$\frac{d\varepsilon}{dt} = \frac{d\varepsilon_2}{dt} + \frac{E_2}{\eta_2}\varepsilon_2 \qquad \text{(S8)}$$

Integrating eq. S7 and S8 and taking into account that dε/dt is constant and at t = 0 ε =0, and t = ε/(dε/dt), for ε_1 and ε_2 eq. S9 and eq. 10 are obtained.

$$\varepsilon_1 = \frac{d\varepsilon}{dt}\frac{\eta_1}{E_1}\left(1 - e^{-\frac{dt}{d\varepsilon}\frac{E_1}{\eta_1}}\right) \qquad \text{(S9)}$$

$$\varepsilon_2 = \frac{d\varepsilon}{dt}\frac{\eta_2}{E_2}\left(1 - e^{-\frac{dt}{d\varepsilon}\frac{E_2}{\eta_2}}\right) \qquad \text{(S10)}$$

Substituting eqs, S9 and S10 into eq. S1, one can get eq. S11

$$\sigma = E\left[\varepsilon + \frac{d\varepsilon}{dt}\frac{\eta_1}{E}\left(1 - e^{-\frac{dt}{d\varepsilon}\frac{E_1}{\eta_1}}\right) + \frac{d\varepsilon}{dt}\frac{\eta_2}{E}\left(1 - e^{-\frac{dt}{d\varepsilon}\frac{E_2}{\eta_2}}\right)\right] \qquad \text{(S11)}$$

where $b_1 = E,\ b_2 = \frac{d\varepsilon}{dt}\frac{\eta_1}{E},\ b_3 = \frac{dt}{d\varepsilon}\frac{E_1}{\eta_1},\ b_4 = \frac{d\varepsilon}{dt}\frac{\eta_2}{E},\ b_5 = \frac{dt}{d\varepsilon}\frac{E_2}{\eta_2}$

Eq. S11 is the same as eq. 12.

6. Dependence of the tensile stress on the relative strain (Figure S16-S28)

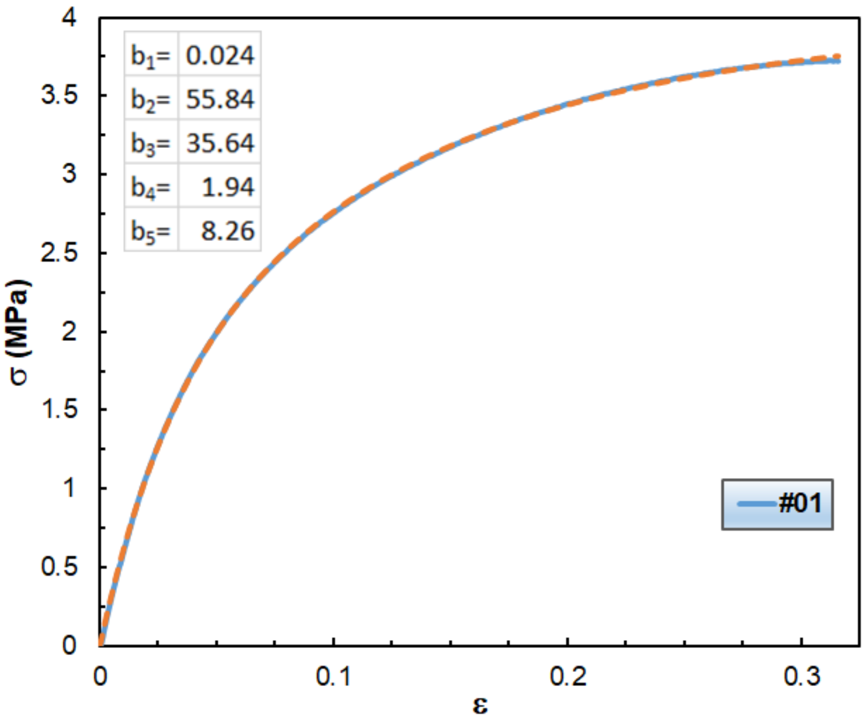

Figure S16. The measured (solid curves) and the fitted (dashed curves) σ *versus* ε data for the sample #01. The fitted parameters are shown in the inset.

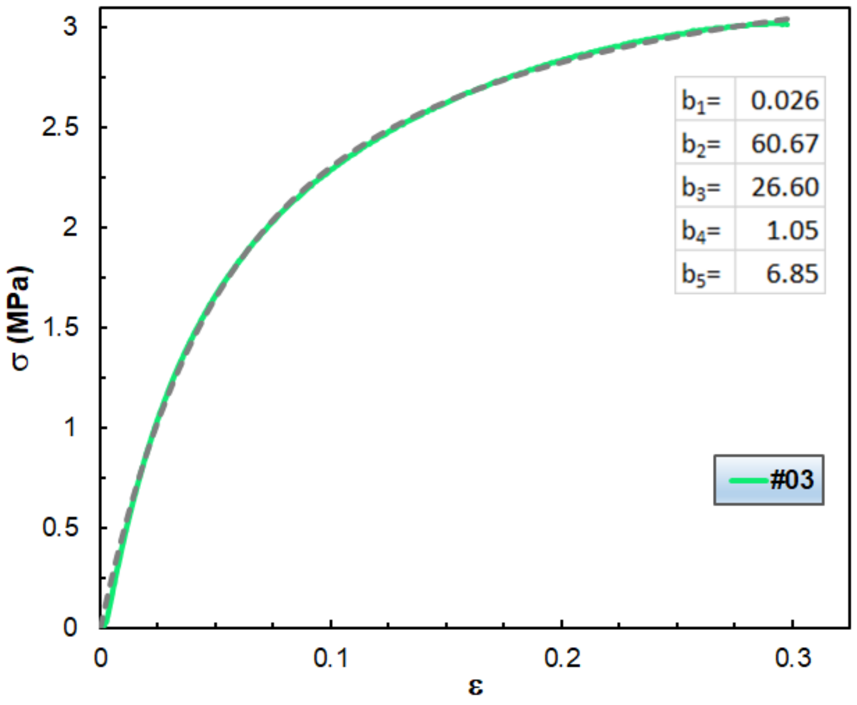

Figure S17. The measured (solid curves) and the fitted (dashed curves) σ *versus* ε data for the sample #03. The fitted parameters are shown in the inset.

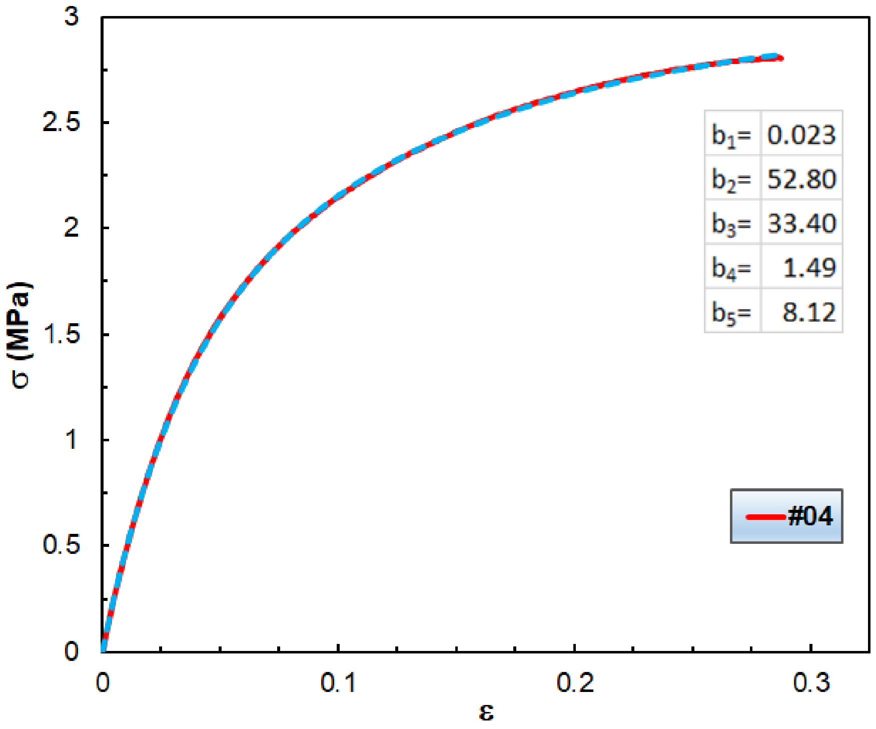

Figure S18. The measured (solid curves) and the fitted (dashed curves) σ *versus* ε data for the sample #04. The fitted parameters are shown in the inset.

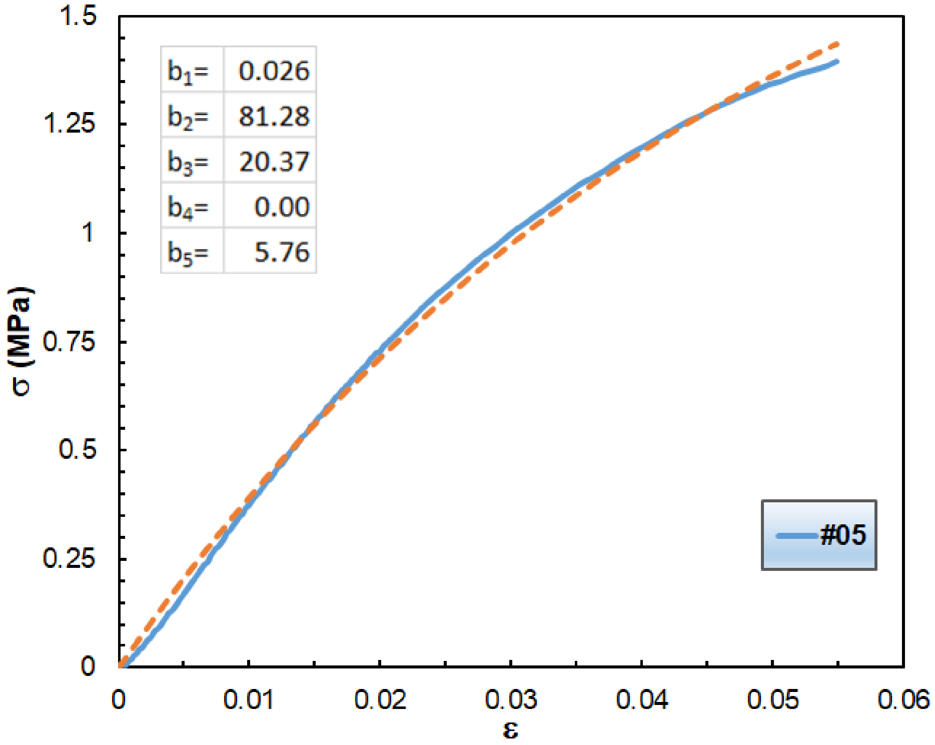

Figure S19. The measured (solid curves) and the fitted (dashed curves) σ *versus* ε data for the sample #05. The fitted parameters are shown in the inset.

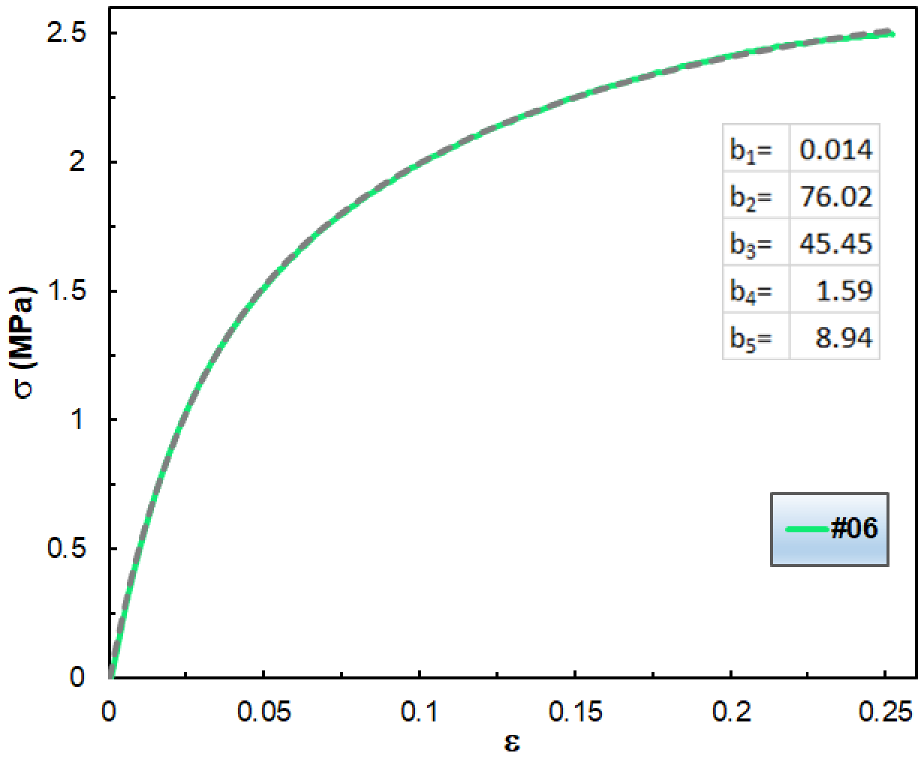

Figure S20. The measured (solid curves) and the fitted (dashed curves) σ *versus* ε data for the sample #06. The fitted parameters are shown in the inset.

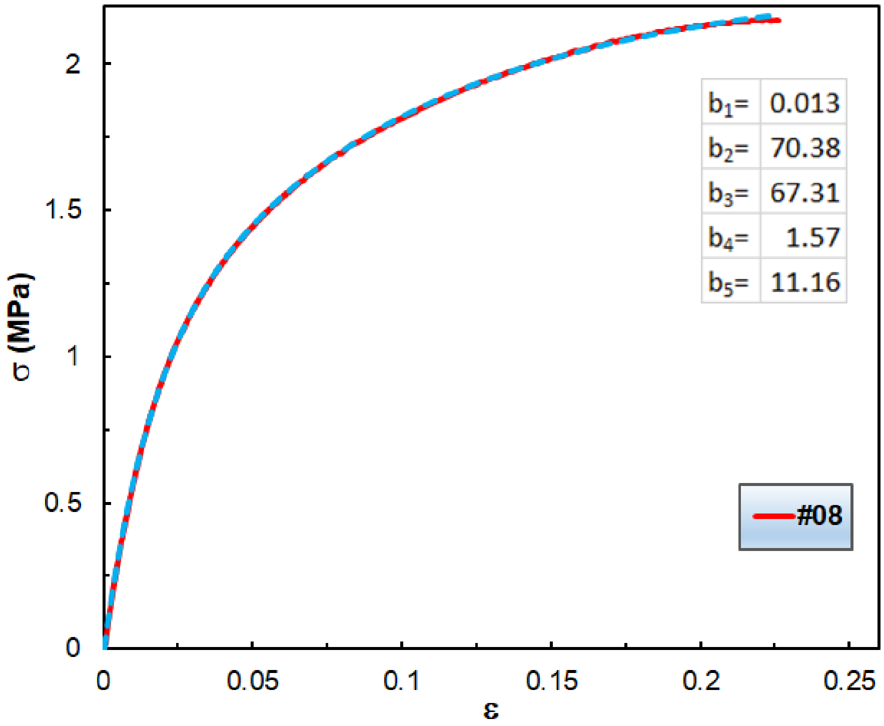

Figure S21. The measured (solid curves) and the fitted (dashed curves) σ *versus* ε data for the sample #08. The fitted parameters are shown in the inset.

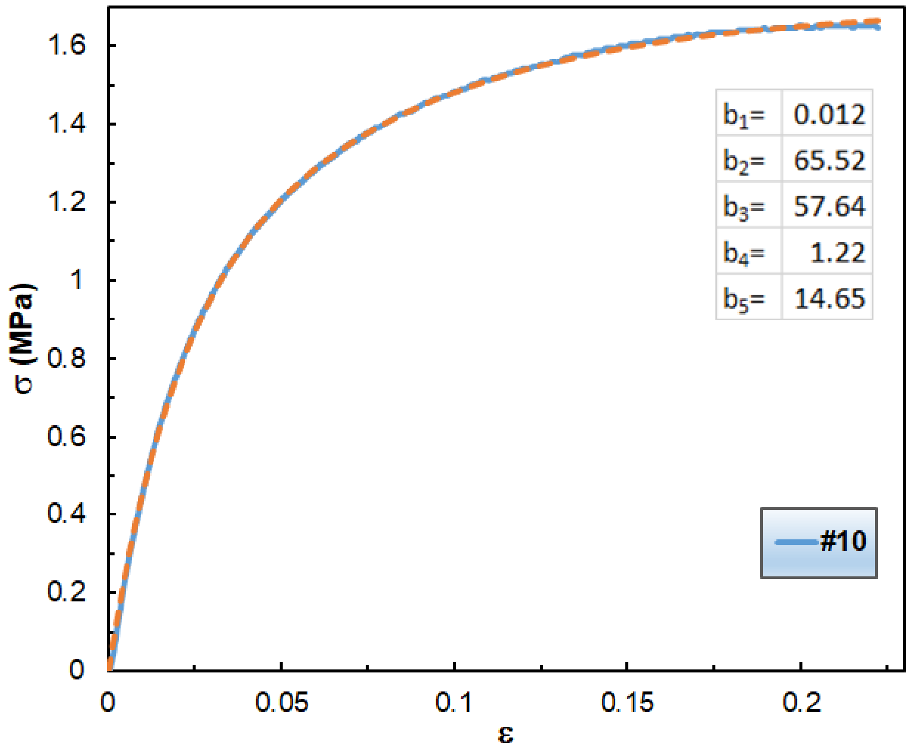

Figure S22. The measured (solid curves) and the fitted (dashed curves) σ *versus* ε data for the sample #10. The fitted parameters are shown in the inset.

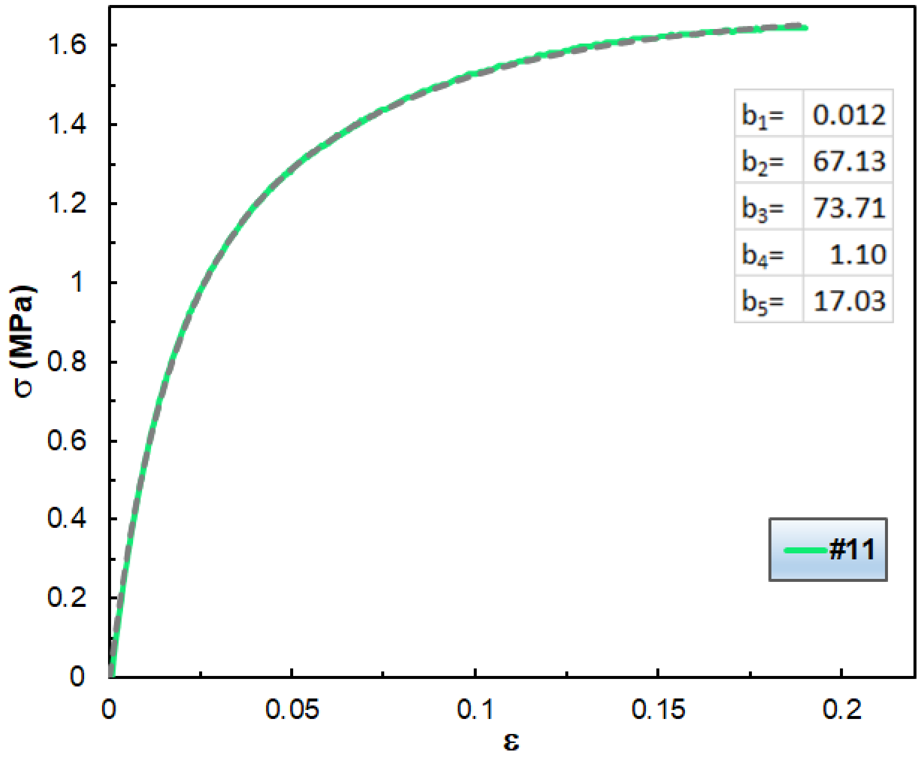

Figure S23. The measured (solid curves) and the fitted (dashed curves) σ *versus* ε data for the sample #11. The fitted parameters are shown in the inset.

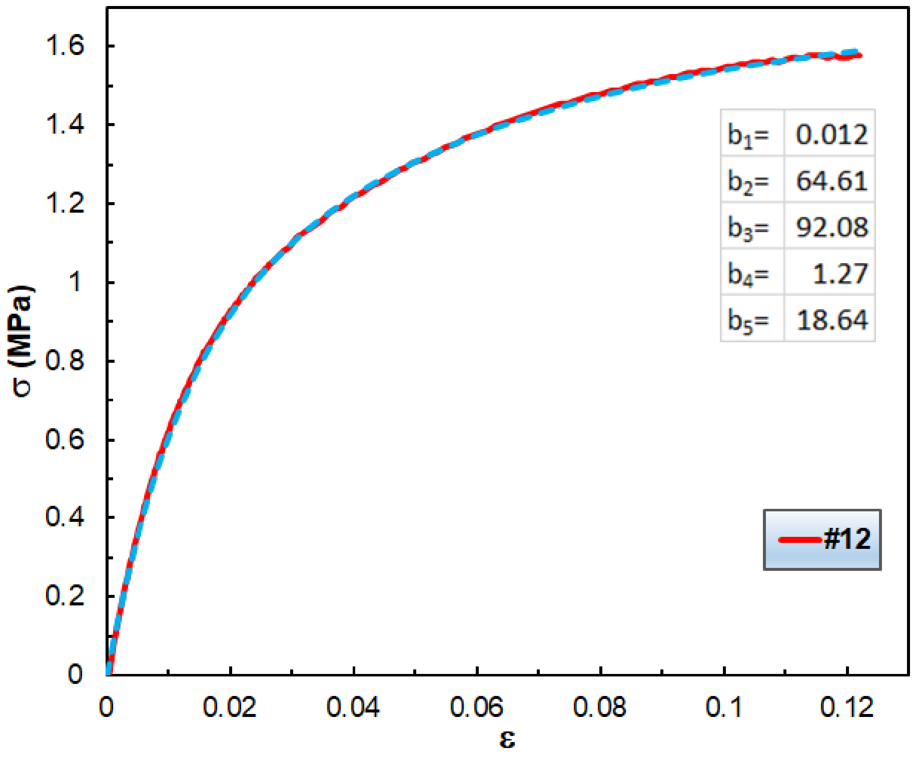

Figure S24. The measured (solid curves) and the fitted (dashed curves) σ *versus* ε data for the sample #12. The fitted parameters are shown in the inset.

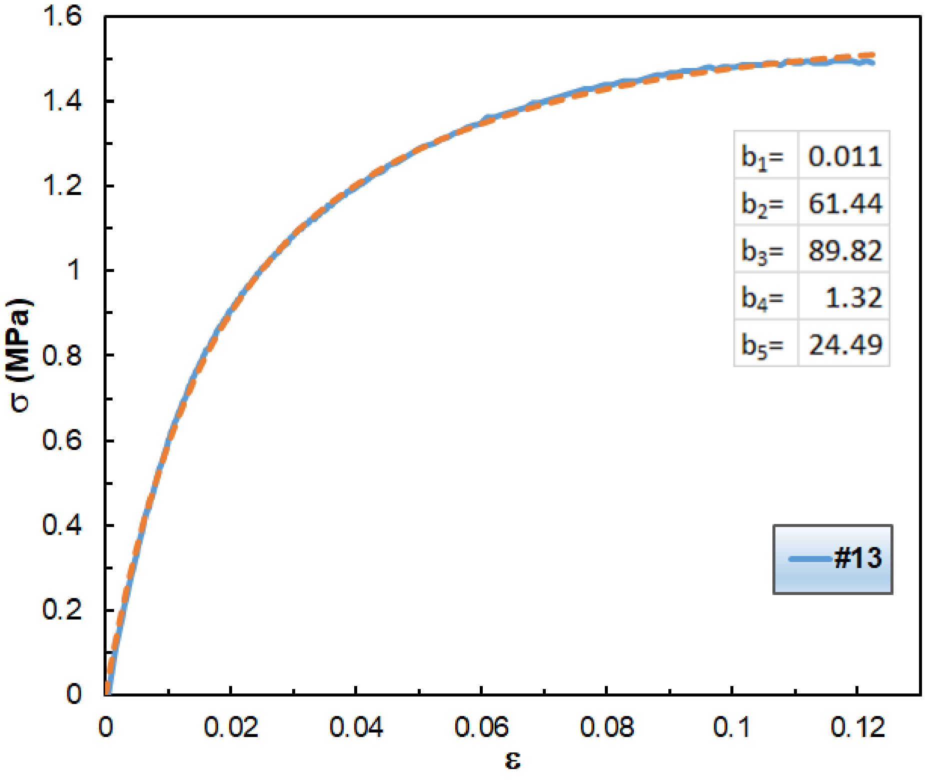

Figure S25. The measured (solid curves) and the fitted (dashed curves) σ *versus* ε data for the sample #13. The fitted parameters are shown in the inset.

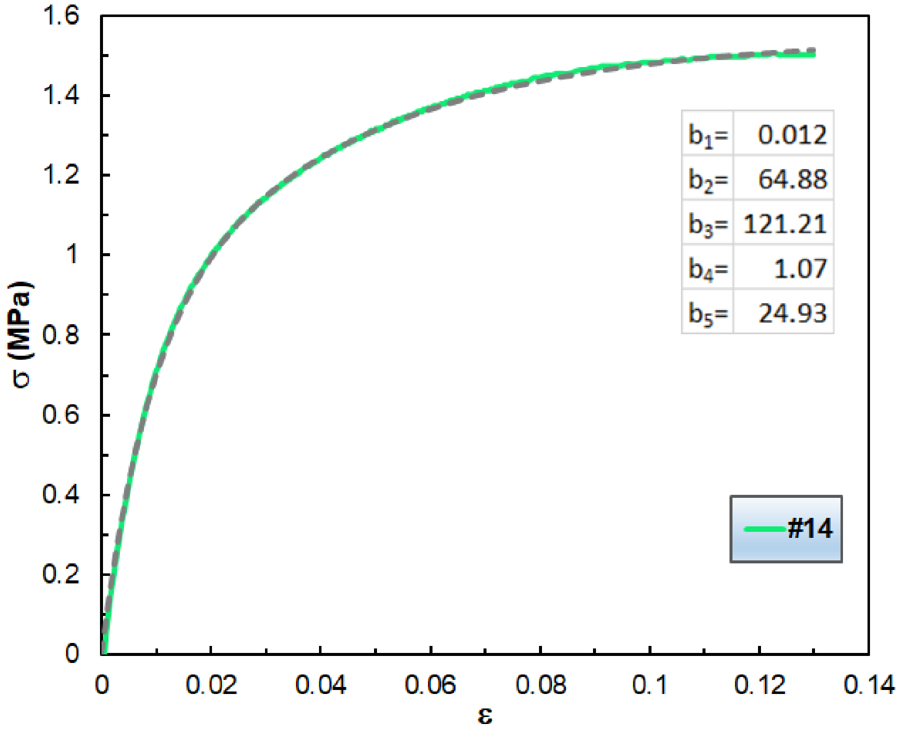

Figure S26. The measured (solid curves) and the fitted (dashed curves) σ *versus* ε data for the sample #14. The fitted parameters are shown in the inset.

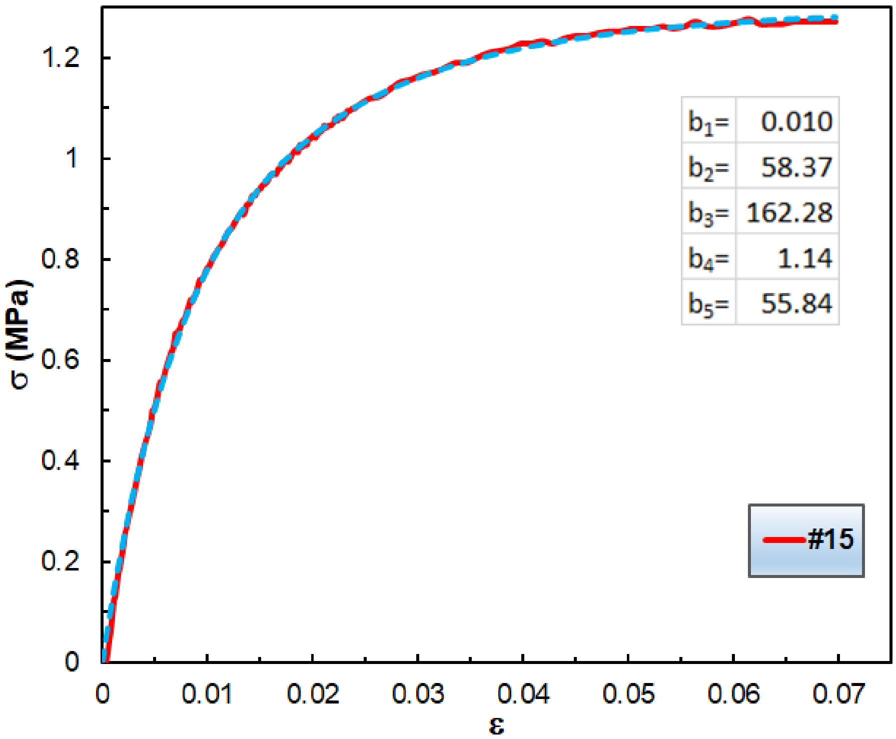

Figure S27. The measured (solid curves) and the fitted (dashed curves) σ *versus* ε data for the sample #15. The fitted parameters are shown in the inset.

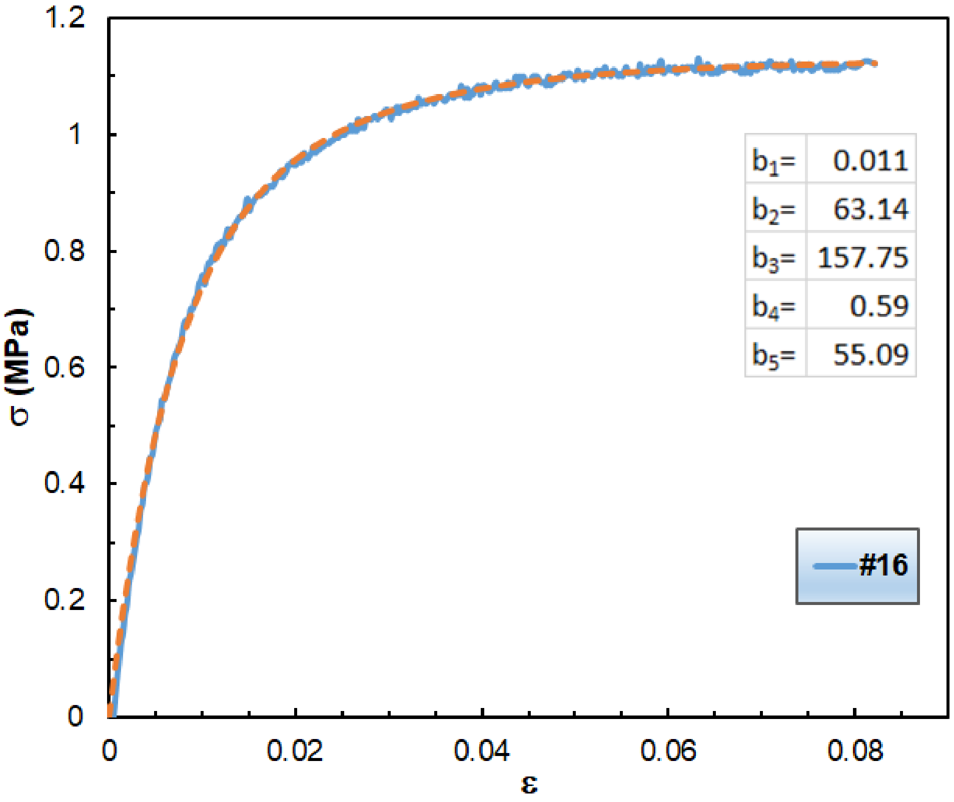

Figure S28. The measured (solid curves) and the fitted (dashed curves) σ *versus* ε data for the sample #16. The fitted parameters are shown in the inset.
